# A *Clostridia*-rich microbiota enhances bile acid excretion in diarrhea-predominant irritable bowel syndrome

**DOI:** 10.1172/JCI130976

**Published:** 2019-12-09

**Authors:** Ling Zhao, Wei Yang, Yang Chen, Fengjie Huang, Lin Lu, Chengyuan Lin, Tao Huang, Ziwan Ning, Lixiang Zhai, Linda L.D. Zhong, Waiching Lam, Zhen Yang, Xuan Zhang, Chungwah Cheng, Lijuan Han, Qinwei Qiu, Xiaoxiao Shang, Runyue Huang, Haitao Xiao, Zhenxing Ren, Dongfeng Chen, Silong Sun, Hani El-Nezami, Zongwei Cai, Aiping Lu, Xiaodong Fang, Wei Jia, Zhaoxiang Bian

**Affiliations:** 1 Institute of Brain and Gut Research, School of Chinese Medicine, Hong Kong Baptist University, Hong Kong SAR, China.; 2 The Second Affiliated Hospital of Guangzhou University of Chinese Medicine, Guangzhou, China.; 3 Shanghai Key Laboratory of Diabetes Mellitus and Center for Translational Medicine, Shanghai Jiao Tong University Affiliated Sixth People’s Hospital, Shanghai, China.; 4 Chinese Medicine Clinical Study Center, School of Chinese Medicine, Hong Kong Baptist University, Hong Kong SAR, China.; 5 School of Pharmaceutical Sciences, Health Science Center, Shenzhen University, Shenzhen, China.; 6 College of Basic Medicine, Guangzhou University of Chinese Medicine, Guangzhou, China.; 7 BGI Genomics, BGI-Shenzhen, Shenzhen, China.; 8 School of Biological Sciences, Faculty of Science, The University of Hong Kong, Hong Kong SAR, China.; 9 School of Chemistry, Hong Kong Baptist University, Hong Kong SAR, China.; 10 Cancer Biology Program, University of Hawaii Cancer Center, Hawaii, USA.

**Keywords:** Gastroenterology, Microbiology, Molecular pathology, Mouse models

## Abstract

An excess of fecal bile acids (BAs) is thought to be one of the mechanisms for diarrhea-predominant irritable bowel syndrome (IBS-D). However, the factors causing excessive BA excretion remain incompletely studied. Given the importance of gut microbiota in BA metabolism, we hypothesized that gut dysbiosis might contribute to excessive BA excretion in IBS-D. By performing BA-related metabolic and metagenomic analyses in 290 IBS-D patients and 89 healthy volunteers, we found that 24.5% of IBS-D patients exhibited excessive excretion of total BAs and alteration of BA-transforming bacteria in feces. Notably, the increase in *Clostridia* bacteria (e.g., *C. scindens*) was positively associated with the levels of fecal BAs and serum 7α-hydroxy-4-cholesten-3-one (C4), but negatively correlated with serum fibroblast growth factor 19 (FGF19) concentration. Furthermore, colonization with *Clostridia*-rich IBS-D fecal microbiota or *C. scindens* individually enhanced serum C4 and hepatic conjugated BAs but reduced ileal FGF19 expression in mice. Inhibition of *Clostridium* species with vancomycin yielded opposite results. *Clostridia*-derived BAs suppressed the intestinal FGF19 expression in vitro and in vivo. In conclusion, this study demonstrates that the *Clostridia*-rich microbiota contributes to excessive BA excretion in IBS-D patients, which provides a mechanistic hypothesis with testable clinical implications.

## Graphical abstract

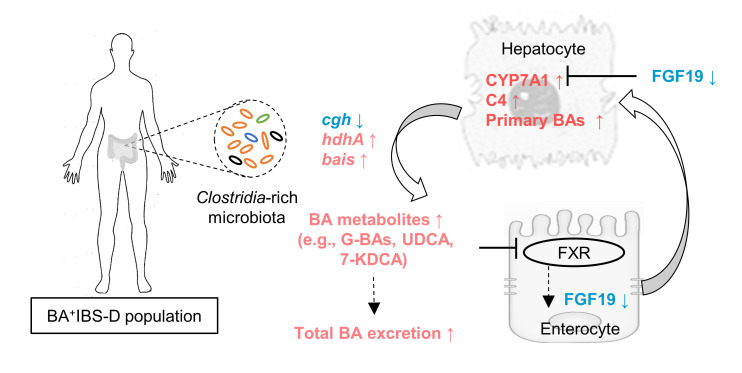

## Introduction

Irritable bowel syndrome (IBS) characterized by irregular bowel habits affects 10% of the worldwide population (1), of which diarrhea-predominant IBS (IBS-D) shows a higher prevalence in Western countries and Asia (2–4). IBS imposes a high economic cost on patients and the government (5). The efficacy of pharmacologic agents for IBS is variable among patients (6, 7), which may be the result of differing etiologies between IBS individuals, as well as imperfect understanding of IBS pathogenesis.

As an important pathogenic factor (8), fecal bile acids (BAs) have been shown to be excessively excreted in 12% to 43% of IBS-D patients (9–12), who are categorized into idiopathic bile acid diarrhea (BAD) (13, 14). Clinical studies have shown that the increase in total fecal BAs is significantly correlated with abdominal pain and accelerated colonic transit in patients (15, 16). Animal studies also showed that BAs directly induce accelerated colonic motility and visceral hypersensitivity (17, 18). Conversely, attenuation of BA excretion with sequestering agents partially alleviated the symptoms of IBS-D patients (19, 20). These observations demonstrate the importance of BA for the development of IBS (21). Nevertheless, how the excessive BA excretion is generated remains incompletely understood.

Normally, BAs are primarily synthesized by the rate-limiting enzyme cholesterol 7α-hydroxylase (CYP7A1) and then conjugated with amino acids in the liver (22). Most conjugated BAs released into the intestine are reabsorbed from the distal ileum and circulate back to the liver via the portal vein, a process known as enterohepatic circulation (22). A small amount of BAs escaping enteric absorption is ultimately excreted in the stool daily, which can be replenished by hepatic de novo synthesis (23). The rate of hepatic BA synthesis is reversibly controlled by farnesoid X receptor–mediated (FXR-mediated) feedback mechanisms in the liver and ileum (24). Moreover, gut microbiota, as an indispensable participant in BA metabolism, are responsible for conversion of primary BAs into secondary BAs in the GI tract (23), as well as interaction with the host for control of BA synthesis (25). Based on current understanding of BA metabolic processes, some host-related mechanisms that contribute to excessive BA excretion have also been proposed as follows: (a) a deficiency of intestine-released feedback regulator fibroblast growth factor 19 (FGF19) (26–28), (b) accelerated regional bowel transit bypassing intestinal BA transport (29), (c) different genotypes in hepatic feedback regulators Klothoβ and FGF receptor 4 (FGFR4) that are closely associated with fecal BAs and colonic transit (11, 12, 30), and (d) upregulation of G protein–coupled BA receptor 1 (GPBAR1) that mediates colonic transit and BA excretion (31).

In addition to dysregulation of host metabolism, alteration of some fecal bacteria has been also reported in IBS-D populations (32, 33). However, the connection between the IBS-D microbiota and BA excretion has received little attention. Given the vital role of commensal microbiota in BA metabolism and the appearance of dysbiosis in the gut ecosystem of IBS-D patients, we hypothesized that altered gut microbiota may contribute to the excessive BA excretion in IBS-D. In this study, by performing BA-related metabolomic and metagenomic analyses in an IBS-D cohort, a *Clostridia*-rich microbiota was identified to be closely associated with excessive BA synthesis and excretion in 25% of IBS-D patients. Further, through a series of animal and cell experiments, we also clarified that the *Clostridia*-rich microbiota can induce BA overexcretion via targeting intestinal feedback control of BA synthesis.

## Results

### Higher levels of BA synthesis in the subgroup of IBS-D patients with excessive BA excretion.

**Figure 1:**
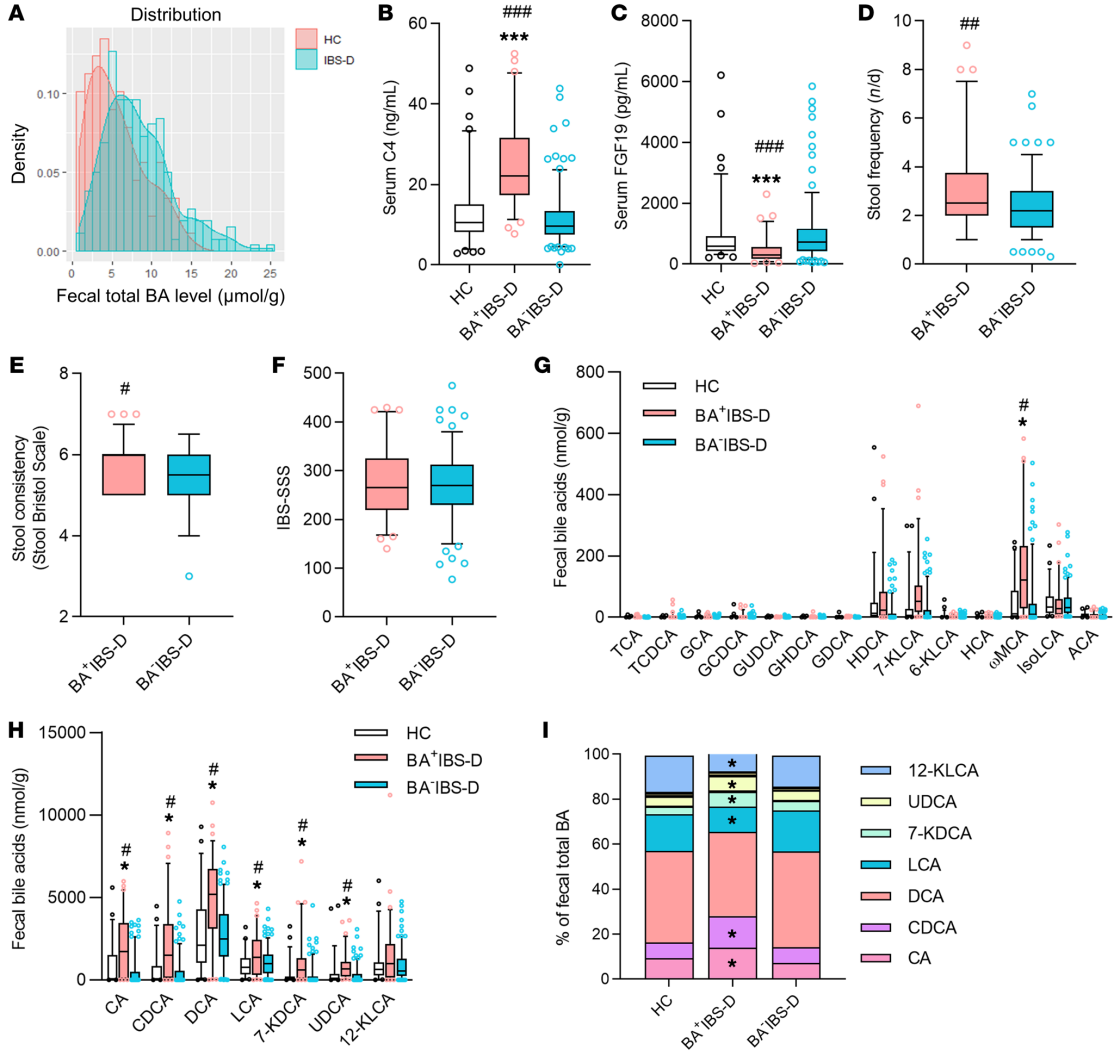
Alteration of fecal BA profiles and serum BA synthetic indicators in IBS-D patients. (**A**) Histogram of the distribution of total fecal BA levels in healthy controls (*n* = 89) and IBS-D patients (*n* = 290). Based on the 90th percentile of healthy total fecal BA level, 25% of IBS-D patients (*n* = 71) with excessive BA excretion were grouped as BA^+^IBS-D and the other patients (*n* = 219) were classified as BA^–^IBS-D. (**B** and **C**) Concentrations of serum 7α-hydroxy-4-cholesten-3-one (C4) and fibroblast growth factor 19 (FGF19). (**D**–**F**) The severity of bowel symptoms between IBS-D subgroups assessed by defecation frequency (**D**), Bristol stool scale (**E**), and IBS severity scoring system (IBS-SSS) (**F**). (**G** and **H**) Absolute contents of fecal dominant BAs. (**I**) Proportions of fecal dominant BAs. Only BAs constituting greater than 1% of the total BA pool are shown in the legend. Differences in phenotypic scores between IBS-D subgroups were analyzed by the Mann-Whitney test, and BA-related indices were evaluated among 3 groups by the Kruskal-Wallis test. The box-and-whisker plots show the mean (horizontal lines), 5th–95th percentile values (boxes), and SEM (whiskers). *P < 0.05, ***P < 0.005 compared with the HC group; ^#^*P* < 0.05, ^##^*P* < 0.01, ^###^*P* < 0.005 compared with the BA^–^IBS-D group. TCA, taurocholic acid; TCDCA, taurochenodeoxycholic acid; GCA, glycocholic acid; GCDCA, glycocheno-deoxycholic acid; GUDCA, glycoursodeoxycholic acid; GHDCA, glycohyodeoxycholic acid; GDCA, glycodeoxycholic acid; CA, cholic acid; CDCA, chenodeoxycholic acid; DCA, deoxycholic acid; LCA, lithocholic acid; 7-KDCA, 7-ketodeoxycholic acid; UDCA, ursodeoxycholic acid; HDCA, hyodeoxycholic acid; KLCA, ketolithocholic acid; HCA, hyocholic acid; ωMCA, ω-muricholic acid; isoLCA, isolithocholic acid; ACA, allocholic acid.

A total of 345 IBS-D adults, defined by predominant bowel habits based on the Bristol stool scale and defecation frequency (34, 35), were recruited at 2 Chinese medical clinics affiliated with the School of Chinese Medicine, Hong Kong Baptist University. Of these participants, 290 patients completed all biochemical tests and consented to voluntarily provide biospecimens (blood and stools) for the study. Referring to the approximately 30% of pooled prevalence of excess BA excretion in the IBS-D population estimated from published studies (9–12), 91 healthy controls (HCs) were recruited and 89 provided biospecimens. As shown in Table 1, the IBS-D cohort displayed a significant increase in defecation frequency and total fecal BAs, whereas it exhibited decreased fecal consistency compared with that of HC subjects. The level of total fecal BA excretion showed a skewed distribution (*P* < 0.05 by the Shapiro-Wilk test) in the IBS-D and HC groups (Figure 1A).

Twenty-five percent of IBS-D patients (71 of 290) were found to have an excess of total BA excretion in feces (≥10.61 μmol/g) by the 90th percentile cutoff value as determined from the HC group. These patients were classified as BA^+^IBS-D. The others with normal fecal BA excretion (<10.61 μmol/g) were grouped as BA^–^IBS-D. Compared with the HC and BA^–^IBS-D groups, BA^+^IBS-D patients also exhibited increased C4 and decreased FGF19 in sera, as well as increased severity of diarrheal symptoms (Table 1 and Figure 1, B–F). Correlation analysis revealed that the total fecal BA levels were positively associated with serum C4 levels and scores of diarrheal symptoms (Bristol stool scale and defecation frequency) but inversely correlated with serum FGF19 levels in the BA^+^IBS-D group (Supplemental Table 1; supplemental material available online with this article; https://doi.org/10.1172/JCI130976DS1). These results demonstrate that enhanced BA synthesis exists in IBS-D patients, accompanied by excessive BA excretion and increased severity of diarrheal symptoms.

Alteration of individual BA levels was also observed in the sera and feces of BA^+^IBS-D patients. Serum BA profiles revealed that glycochenodeoxycholic acid (GCDCA), glycoursodeoxycholic acid (GUDCA), and chenodeoxycholic acid (CDCA) were significantly elevated in both absolute amounts and relative proportions in BA^+^IBS-D patients compared with those of the HC group (Supplemental Figure 1). In addition, BA^+^IBS-D patients had an increased absolute level of ursodeoxycholic acid (UDCA) and a reduced relative proportion of glycohyodeoxycholic acid (GHDCA) in sera. The fecal BA pool of all recruits was largely composed of free BAs, as previously described (36), of which cholic acid (CA), CDCA, deoxycholic acid (DCA), lithocholic acid (LCA), 7-ketodeoxycholic acid (7-KDCA), UDCA, and ω-muricholic acid (ωMCA) showed significant increases in their absolute amounts in the BA^+^IBS-D group compared with the HC group (Figure 1, G and H). Meanwhile, the proportions of CA, CDCA, UDCA, and 7-KDCA increased in total fecal BAs, whereas the proportions of LCA and 12-KLCA decreased in the BA^+^IBS-D group (Figure 1I). In addition, among the abundant conjugated BAs, GCDCA was slightly increased in feces of BA^+^IBS-D patients, while GUDCA showed no difference. However, BA^–^IBS-D patients showed similar serum and fecal BA compositions as those of the HC subjects. These metabolic results reveal an altered composition of microbiota-derived BAs (e.g., GUDCA, UDCA, 7-KDCA, etc.) in sera and feces of BA^+^IBS-D patients, indicating that an abnormality in BA-transforming gut microbiota might contribute to the cause of this condition.

### Association of a Clostridia-rich microbiota with increased BA synthesis and excretion in BA^+^IBS-D patients.

**Figure 2:**
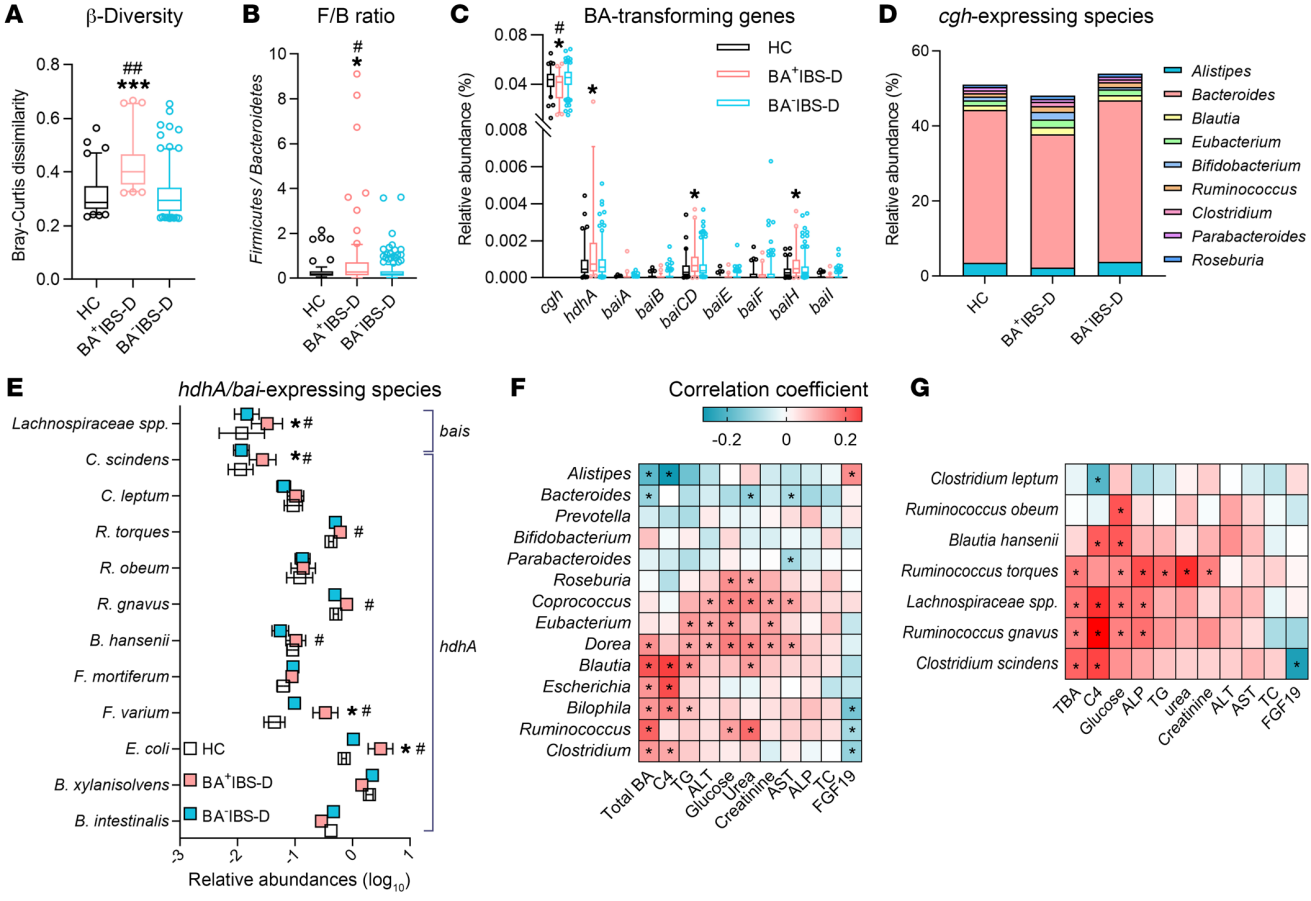
The association between *Clostridia*-rich microbiota and the levels of BA synthesis and excretion in the IBS-D cohort. (**A**) Microbial β-diversities measured by Bray-Curtis dissimilarity. (**B**) The ratio of *Firmicutes* to *Bacteroidetes* (F/B). (**C**–**E**) The relative abundances of BA-transforming genomes and bacteria. (**F** and **G**) Spearman’s correlation between bacterial abundances and biochemical indices in the IBS-D cohort. The metagenomic data set was obtained from 277 IBS-D and 84 HC fecal samples. Differential taxa and genes among 3 groups were analyzed with the Benjamin-Hochberg method. The box-and-whisker plots show the mean (horizontal lines), 5th–95th percentile values (boxes), and SEM (whiskers). *P < 0.05, ***P < 0.005 compared with the HC group; ^#^*P* < 0.05; ^##^*P* < 0.01 compared with the BA^–^IBS-D group. Statistical significance for Spearman’s correlation was defined as *P* < 0.05. ALT, alanine aminotransferase; TG, triglycerides; AST, aspartate aminotransferase; ALP, alkaline phosphatase; TC, total cholesterol; *cgh*, gene coding choloylglycine hydrolase; *hdhA*, gene encoding 7α-hydroxysteroid dehydrogenase; *bai* *A/B/CD/E/F/H/I*, BA-inducible genes *A/B/CD/E/F/H/I*; *C*. *scindens*, *Clostridium scindens*; *C*. *leptum*, *Clostridium leptum*; *R*. *torques*, *Ruminococcus torques*; *R*. *obeum*, *Ruminococcus obeum*; *R*. *gnavus*, *Ruminococcus gnavus*; *B*. *hansenii*, *Blutia hansenii*; *F*. *mortiferum*; *Fusobacterium mortiferum*; *F*. *varium*, *Fusobacterium varium*; *E.coli*, *Escherichia coli*; *B*. *xylanisolvens*, *Bacteroides xylanisolvens*; *B*. *intestinalis*, *Bacteroides intestinalis*.

Fecal metagenomic data were successfully obtained from 84 HC subjects, 70 BA^+^IBS-D patients, and 207 BA^–^IBS-D patients. An average of 6.04 gigabases of high-quality sequencing reads was obtained from each sample, and an average of 63.7% reads per sample were successfully mapped (Supplemental Table 2). In comparison with the HC and BA^–^IBS-D groups, fecal microbial communities exhibited a higher Bray-Curtis dissimilarity in the BA^+^IBS-D group, but without differences in total gene count and Shannon index (Figure 2A and Supplemental Figure 2, A and B). These results indicate the unchanged microbial richness within all included subjects, but a larger instability of the enteric ecosystem in the BA^+^IBS-D subgroup.

Principal coordinate analysis revealed an overlap of fecal microbial communities among IBS-D and HC subjects (Supplemental Figure 2C); however, a different microbial profile was found in BA^+^IBS-D patients in comparison with either HC or BA^–^IBS-D subjects at different taxonomic levels (Supplemental Tables 3–5). Specifically, the relative abundances of the phyla *Firmicutes*, *Actinobacteria*, *Fusobacteria*, and *Proteobacteria* were increased, while that of *Bacteroidetes* was decreased in BA^+^IBS-D patients (Supplemental Figure 2D). Accordingly, the ratio of *Firmicutes* to *Bacteroidetes* was significantly elevated (Figure 2B). At the genus level, the abundances of *Clostridia* bacteria, including *Ruminococcus*, *Clostridium*, *Eubacterium*, and *Dorea* were significantly increased in BA^+^IBS-D fecal microbiota (Supplemental Figure 2E). The abundances of *Bifidobacterium*, *Escherichia*, and *Bilophila* were also increased. However, the abundances of *Alistipes* and *Bacteroides* were reduced significantly compared with those of HC or BA^–^IBS-D fecal microbial communities.

The alteration in the bacterial composition of the BA^+^IBS-D group was also associated with variation in BA-transforming genomes (Figure 2C and Supplemental Table 6). Reduced abundances of *Alistipes* and *Bacteroides* were mainly associated with a decreased abundance of the *cgh* gene (Figure 2D), which encodes the BA-deconjugating enzyme choloylglycine hydrolase (36). However, an elevated abundance of the *hdhA* gene, encoding 7α-hydroxysteroid dehydrogenase (7α-HSDH) (36), was attributed to increases in *Escherichia*, *Fusobacterium*, *Blautia*, *Ruminococcus*, and *Clostridium* species (Figure 2E). Moreover, *Clostridium scindens* and an unclassified *Lachnospiraceae* species largely contributed to higher abundances of the genes *baiCD* and *baiH* (Figure 2E), which are known to encode 7-dehydroxylases (36). Correlation analysis revealed that the abundances of *Clostridia* genera and *C*. *scindens* species were positively correlated with the concentrations of total fecal BAs and serum C4, but negatively correlated with the serum FGF19 level (Figure 2, F and G). These results suggest a specific *Clostridia*-rich microbiota in the BA^+^IBS-D group, with different genomes for BA deconjugation, C7 isomerism, and dehydroxylation. Given the importance of gut microbiota in maintenance of host BA metabolism (22), the significant associations between gut bacteria and BA indices in the IBS-D cohort suggest the possibility that the *Clostridia*-rich microbiota may influence patients’ BA synthesis and excretion.

### Enhancement of BA synthesis and excretion in pseudo-germ-free mice transplanted with Clostridia-rich fecal microbiota.

**Figure 3:**
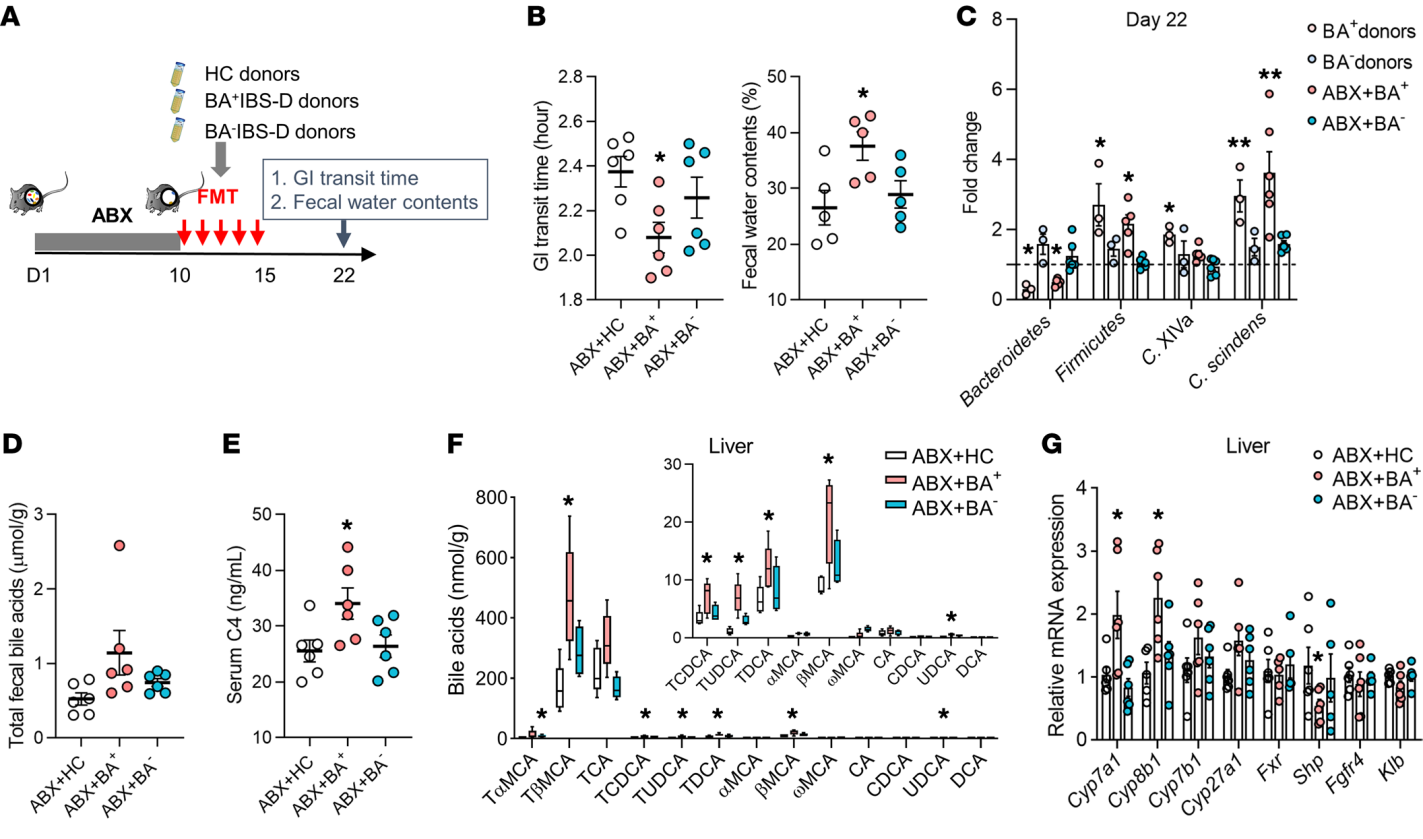
Excessive BA synthesis and excretion in mouse recipients receiving BA^+^IBS-D fecal microbiota. (**A**) Experimental procedure for fecal microbiota transplantation (FMT) in antibiotic cocktail–induced (ABX-induced) pseudo-germ-free mice (*n* = 6/group). Mice that received fecal microbiota of HC donors were grouped as ABX+HC, and mice treated with fecal microbiota from BA^+^IBS-D and BA^–^IBS-D donors were classified as ABX+BA^+^ and ABX+BA^–^, respectively. (**B**) The GI transit time and fecal water contents of mouse recipients. (**C**) Relative levels of BA-related bacteria in feces of donors and mouse recipients based on qPCR analysis. (**D** and **E**) The levels of total fecal BAs and serum C4 in mouse recipients. (**F**) Hepatic BA profiles of mouse recipients. (**G**) Relative gene expression of BA synthetic regulators in the hepatic tissues of mouse recipients. Differential BA-related phenotypes, bacteria, and genes are shown as mean ± SEM. BA metabolites are expressed with 5th–95th percentile values. Differences were assessed with the Kruskal-Wallis test. *P < 0.05, **P < 0.01 compared with the ABX+HC group. *Cyp7a1*, *Cyp8b1*, *Cyp7b1*, *Cyp27a1*, *Fxr*, *Shp*, *Fgfr4*, and *Klb* represent the mRNAs for the proteins cholesterol 7α-hydroxylase, sterol 12α-hydroxylase, steroid 7α-hydroxylase, sterol 27-hydroxylase, farnesoid X receptor, small heterodimer partner, fibroblast growth factor receptor 4, and Klothoβ, respectively.

To investigate the effects of the *Clostridia*-rich microbiota on BA synthesis and excretion, transplantation of BA^+^IBS-D fecal microbiota to pseudo-germ-free mice was conducted (Figure 3A and Supplemental Figure 3A). One week after microbial transplantation, BA^+^IBS-D microbiota mouse recipients displayed shortened GI transit time and increased fecal water content (Figure 3B) that were similar to the diarrheal symptoms of their donors. Fecal profiles of BA-transforming bacteria in recipients were similar to those of BA^+^IBS-D donors, with a reduced abundance of *Bacteroidetes* and elevated abundances of *Firmicutes*, *Clostridium* cluster XIVa, and *C*. *scindens* (Figure 3C). An in vitro transforming-activity assay with detection of the ratio of BA products to substrates found that the cecal microbiota isolated from mouse recipients exhibited a decreased deconjugating capability but a slight increase in 7-HSDH and 7α-dehydroxylation (Supplemental Figure 3B). These results are consistent with the BA-transforming activities in the fecal microbiota of the human BA^+^IBS-D donors (Supplemental Figure 3C).

Metabolic analysis showed increases in total fecal BAs and serum C4 in the BA^+^IBS-D microbiota recipients, along with elevated taurine-conjugated BAs (TβMCA, TCA, TCDCA, and TUDCA) in the liver and ileal lumen (Figure 3, D–F and Supplemental Figure 3D). Furthermore, tissue mRNA analysis showed increased expression of hepatic *Cyp7a1* and *Cyp8b1* but reduced expression of hepatic *Shp* and ileal *Fgf15* in BA^+^IBS-D microbiota recipients (Figure 3G and Supplemental Figure 3E). Increased expression of hepatic CYP7A1 and decreased expression of ileal FGF15 were also confirmed at the protein level (Supplemental Figure 3F). Additionally, the mRNA analysis of hepatic FGF19/15 receptor complexes (*FGFR4* and Klothoβ [*KLB*]) and ileal BA active transporters (apical sodium bile acid transporter, *ASBT*; multidrug resistance–associated protein 2 and 3 (*MRP2* and -*3*); and organic solute transporter α/β (*OSTA* and -*B*) showed no changes among recipient groups when compared with those of HC microbiota recipients (Figure 3G and Supplemental Figure 3E). These results demonstrate that the *Clostridia*-rich microbiota of BA^+^IBS-D donors could induce diarrhea-like phenotypes and enhance BA synthesis and excretion in mouse recipients, but these effects are probably independent of ileal BA absorption and hepatic feedback inhibition.

### Enhancement of hepatic BA synthesis and excretion in mice with colonization of Clostridium species.

**Figure 4:**
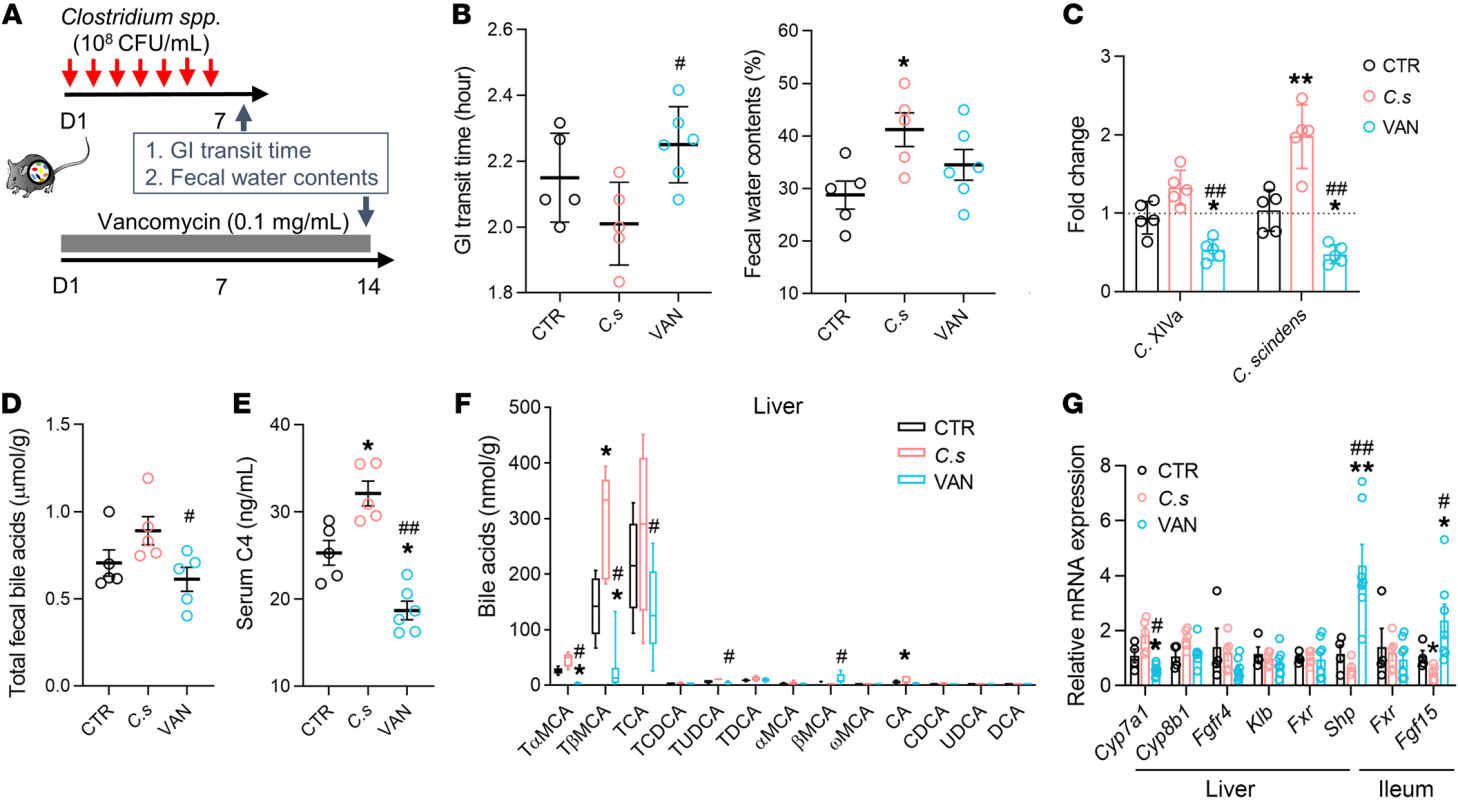
Dysregulation of BA synthesis and excretion in mice with manipulation of *Clostridium* species. (**A**) Manipulation of *Clostridium* species in conventional mice (*n* = 5–6/group) through introduction of *C*. *scindens* strain (*C. s*) or administration of vancomycin (0.1 mg/mL). (**B**) The GI transit time and fecal water contents of *Clostridium*-treated mice. (**C**) Relative levels of the fecal *Clostridial* bacteria (*C*. XIVa) and *C*. *scindens* measured by qPCR analysis. (**D** and **E**) The levels of total fecal BAs and serum C4. (**F**) The BA profile in the mouse liver. (**G**) Relative gene expression of BA synthetic regulators in the hepatic and ileal tissues. Differential BA-related phenotypes, bacteria, and genes are shown as mean ± SEM. BA metabolites are expressed with 5th–95th percentile values. Statistical significance was determined with the Kruskal-Wallis test. *P < 0.05, **P < 0.01 compared with the control group; ^#^*P* < 0.05, ^##^*P* < 0.01 compared with the *C*. *scindens* group. CTR, control.

To further clarify the effects of *Clostridium* species on BA synthesis and excretion, we introduced the *C*. *scindens* strain (1 × 10^8^ CFU/mL) and specifically inhibited *Clostridium* species using vancomycin (0.1 mg/mL) in 2 separate groups of conventional mice (Figure 4A). Colonization of *C*. *scindens* significantly decreased the fecal consistency in mice when compared with mice treated with vehicle. Vancomycin treatment significantly attenuated the GI transit (Figure 4B). Moreover, qPCR analysis showed a significant increase in *C*. *scindens* abundance in the cecum of *C*. *scindens*–colonized mice compared with that of controls (Figure 4C). The transforming-activity assay showed a slightly reduced deconjugating activity and a significantly increased activity of 7-HSDH in the cecal microbiota of *C*. *scindens* recipients (Supplemental Figure 4A). In contrast, the abundances of *Clostridium* species in the cecum of vancomycin-treated mice significantly decreased, along with the reduction of in vitro activities of 7-HSDH and 7-dehydroxylases, and an increase in BA deconjugating capability (Figure 4C and Supplemental Figure 4A).

Metabolic analysis found that the total fecal BA and serum C4 concentration significantly increased in *C*. *scindens*–colonized mice, while these were reduced in vancomycin-treated mice (Figure 4, D and E). Consistently, the concentrations of taurine-conjugated BAs (TβMCA, TCA, and TUDCA) were significantly increased in the liver and the ileal lumen of *C*. *scindens*–colonized mice, but were significantly reduced in vancomycin-treated mice (Figure 4F and Supplemental Figure 4B). Hepatic *Cyp7a1* mRNA expression was elevated in *C*. *scindens*–colonized mice, but significantly reduced in vancomycin-treated mice (Figure 4G). *C*. *scindens* colonization significantly attenuated *Fgf15* expression in the ileum, which was also verified at the protein level (Figure 4G and Supplemental Figure 4C). However, there was no difference in the expression of *Fgfr4* and *Klb* among the groups (Figure 4G). Although *Fxr* expression showed no difference within groups with bacterial manipulation, hepatic *Shp* and ileal *Fgf15* genes were dramatically increased in vancomycin-treated mice. Furthermore, we also found that treatment with either vancomycin (from 10 to 400 μM) or *C*. *scindens* strains (live and heat-killed) had no direct effect on the expression of *CYP7A1* in L-02 hepatocytes (Supplemental Figure 4, D and E). These findings suggest that the effects of *Clostridium* species on enhanced BA excretion and diarrhea-like phenotypes are involved in FXR-mediated feedback control of BA synthesis.

### Inhibitory effects of Clostridia-derived BAs on the intestinal negative feedback signaling.

Given the inhibitory impact of TβMCA on FXR activation (25), we proposed that the excess of taurine-conjugated BAs (TCA, TCDCA, and TUDCA), consistently detected in mice colonized with *Clostridia*-rich microbiota and *C*. *scindens*, can inhibit FXR-mediated feedback signaling. To verify this notion, the effects of TCA, TCDCA, TUDCA, and their mixture (T-BAs) on hepatic and intestinal FXR feedback pathways were examined in vivo and in vitro.

**Figure 5:**
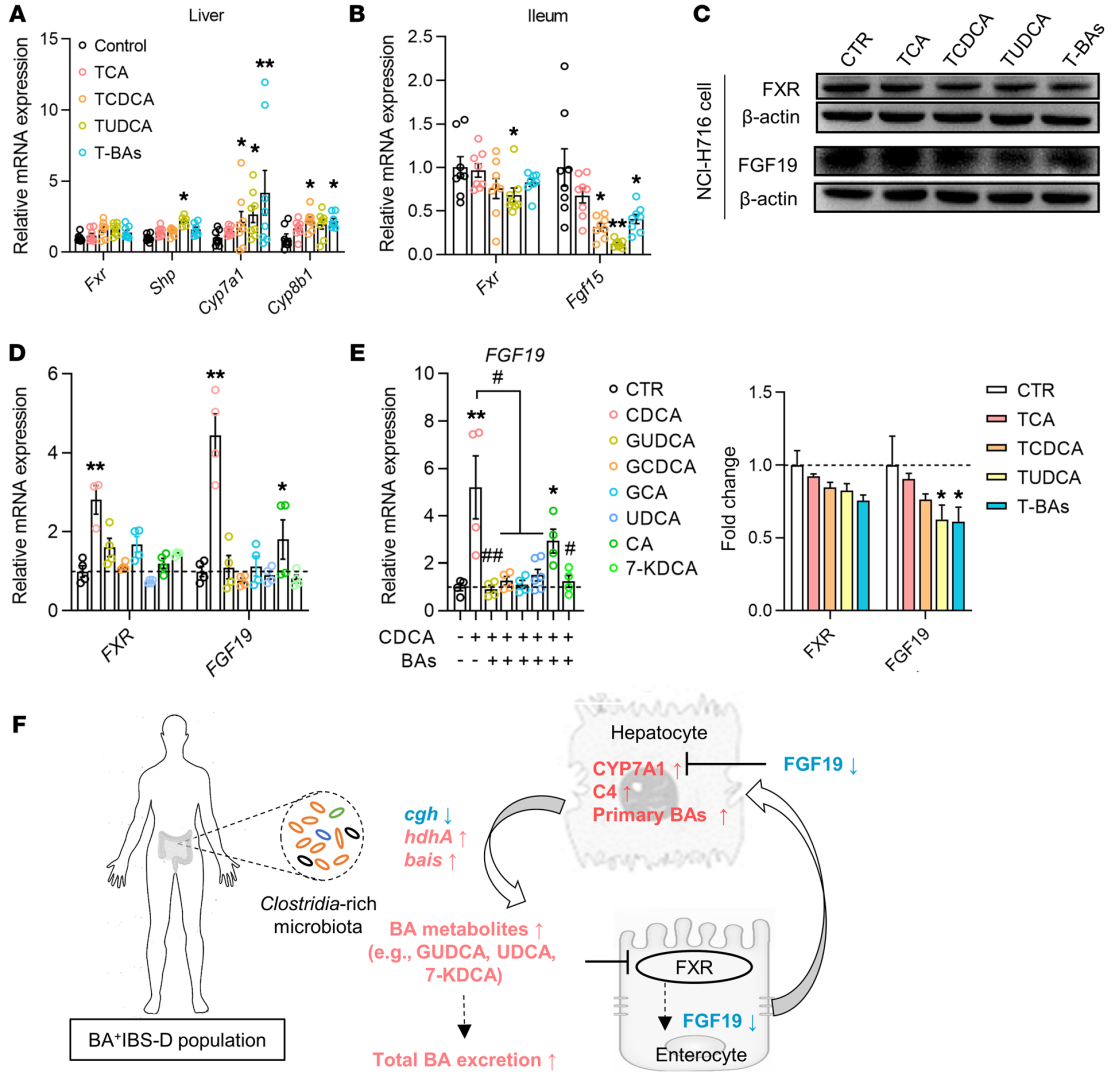
The inhibitory effects of *Clostridia*-derived BAs on intestinal FXR feedback signaling. (**A** and **B**) Hepatic and ileal expression of the FXR gene and its target genes in mice with intervention of taurine-conjugated BAs (*n* = 8/group). (**C**) Western blot showing expression of FXR and FGF19 in enterocytes treated with BA-taurine conjugates that were derived from mouse *Clostridia*-rich microbiota. T-BAs, combination of TCA, TCDCA, and TUDCA. (**D**) Gene expression of FXR and FGF19 in enterocytes treated with individual BAs that were derived from human *Clostridia*-rich microbiota. (**E**) Gene expression of FGF19 in enterocytes treated with combinations of the FXR agonist CDCA and each *Clostridia*-derived BA. (**F**) Schematic diagram of a potential mechanism by which the *Clostridia*-rich microbiota contribute to excessive BA synthesis and excretion in BA^+^IBS-D. Differential proteins and genes are presented as mean ± SEM and were analyzed with the Kruskal-Wallis test. *P < 0.05, **P < 0.01 compared with the control group; ^#^*P* < 0.05, ^##^*P* < 0.01 compared with the CDCA group. CTR, control.

We first tested individual effects of TCA, TCDCA, TUDCA, and T-BAs (50 mg/kg/d for each) on the FXR-mediated feedback system in mice. Compared with the control group with saline treatment, TUDCA reduced ileal *Fxr* gene expression in mice. TCDCA, TUDCA, and T-BAs decreased mouse ileal *Fgf15* expression but elevated hepatic *Cyp7a1* expression (Figure 5, A and B). All 4 BA treatments had no effect on hepatic *Fxr* expression but TUDCA increased *Shp* expression in the liver (Figure 5A). We also confirmed effects of TCA, TCDCA, TUDCA, and T-BAs on FXR signaling in vitro. Referring to a published EC_50_ range for FXR ligands (37), we found that 50 μM TUDCA and T-BAs both significantly reduced FGF19 expression in NCI-H716 enterocytes (Figure 5C), which is similar to in vivo observations. Additionally, 50 μM TUDCA reduced FXR and small heterodimer partner (SHP) expression in L-02 hepatocytes but significantly elevated CYP7A1 expression (Supplemental Figure 5). These results revealed that TUDCA inhibited intestinal FXR/FGF15/19 signaling in vivo and in vitro, but showed inconsistent effects on hepatic FXR/SHP signaling that need to be further investigated.

Abundant BAs (GCDCA, GUDCA, GCA, CDCA, CA, UDCA, and 7-KDCA) detected in serum or the fecal BA pool of BA^+^IBS-D patients were used to examine effects on FXR and FGF19 in enterocytes. As potent natural agonists of FXR (38), CDCA and CA activated FXR and dramatically elevated *FGF19* expression (Figure 5D). Other BAs (GCDCA, GUDCA, GCA, UDCA, and 7-KDCA) had no effect on FXR, but could efficiently antagonize CDCA-induced FXR activation (Figure 5, D and E). Collectively, the in vivo and in vitro results showing that *Clostridia*-derived BAs, particularly conjugated and free UDCA, attenuate intestinal FGF19/15 production suggest that *Clostridia*-rich microbiota and *C*. *scindens* could inhibit intestinal negative feedback signaling.

## Discussion

This study identified a *Clostridia*-rich microbiota with imbalanced BA-transforming activity in BA^+^IBS-D patients. *Clostridia*-rich gut dysbiosis has strong correlation with the increased BA synthesis/excretion in the IBS-D cohort. Transplantation with *Clostridia*-rich fecal microbiota from BA^+^IBS-D patients or colonization of *Clostridium* species significantly enhanced BA synthesis and excretion in mouse recipients. Mechanistic experiments revealed that *Clostridia*-derived BAs attenuate the intestinal BA feedback inhibition.

An excess of BA delivery in the GI lumen is an important pathogenic factor for IBS-D (21). Given the previously reported stimulatory impact of free primary and secondary BAs on GI motor and/or secretory function (39, 40), the increase in excreted free BAs (particularly CDCA, DCA, LCA, and UDCA) likely contributes to the diarrheal phenotype in BA^+^IBS-D patients. To explore the cause of the excessive BAs, previous studies with IBS-D or functional diarrhea have reported increased C4 and/or decreased FGF19 concentrations in the sera of patients (12, 19, 27, 41, 42) together with impaired basal and stimulated FGF19 expression in ileal biopsies (28). These results lead to the hypothesis that excessive BAs of IBS-D results from the deficiency of FGF19 (43, 44). Nevertheless, until now, the reasons for the deficiency of FGF19 have not been fully explained. Previous studies found an unchanged genotype and mRNA expression of the BA transporter ASBT in ileal biopsies, and similar BA-transport activities excluded the contribution of ileal absorption to excessive BAs in IBS-D or chronic diarrheal patients (11, 45, 46). Our study found that there is a remarkable increase in GCDCA and GUDCA in the sera of BA^+^IBS-D patients but not in the feces, together with the raised UDCA levels in the sera and feces. Considering that there are multiple mechanisms for transporting BAs with and without conjugated amino acids across the intestinal membrane (47), our data indicate that the ileal malabsorption should be irrelevant to the excessive BAs in BA^+^IBS-D patients.

The serum and fecal BA profiles in BA^+^IBS-D patients revealed that the microbiota-derived BAs are significantly increased, indicating there might be an abnormality in BA-transforming gut microbiota. Fecal metagenomic data support the alteration in the BA-transforming bacterial composition, specifically the presence of *Clostridia*-rich gut microbiota, among BA^+^IBS-D patients. The BA-transforming genome results also support this assumption. Notably, transplantation of *Clostridia*-rich gut microbiota from BA^+^IBS-D patients and colonization of *C*. *scindens* species can induce altered stool in mice, which is similar to the clinical phenotypes of IBS-D patients. The transplantation also resulted in an increase in total fecal BAs and excessive BA synthesis in the liver, accompanying the defective ileal FGF15 expression and an increase in C4 in sera. These changes are similar to the excessive BA excretion, increased C4, and decreased FGF19 expression in BA^+^IBS-D patients. These findings indicate a gut microbiota–driven mechanism, specifically *Clostridia*-rich microbiota-driven excessive BA synthesis and excretion.

BA biotransformation by gut microbiota has received relatively little attention in IBS-D. First, our in vitro transforming-activity assay revealed a reduced deconjugating activity of isolated gut microbiota from IBS-D patients, which is similar to previous reports (15, 48). In contrast with previous studies, however, the lower deconjugating capability was specifically shown in BA^+^IBS-D subjects rather than BA^–^IBS-D patients in our study. Also, human omics data showing that the increased proportions of serum GUDCA together with decreased abundances of fecal *cgh* and *cgh*-expressing genera in BA^+^IBS-D patients indicate a lower deconjugating activity for the *Clostridia*-rich microbiota. Further, the decrease in in vitro deconjugating activity of cecal microbiota isolated from mouse recipients and the increase in taurine-conjugated BAs in mouse ileal lumen also indirectly support the decreased deconjugating capacity. These data support the findings of increased conjugated BAs in the BA^+^IBS-D patients. Second, gut microbiota from the BA^+^IBS-D patients was also characterized by the enrichment of *hdhA/bai*-expressing *Clostridium* species (e.g., *C*. *scindens*) along with the elevated in vitro activities of 7-HSDH and 7-dehydroxlases. Marion et al. revealed that, apart from 7α-dehydroxylation, *C*. *scindens* species also oxidize other hydroxyl groups and reduce ketone groups in primary and secondary bile acids in vitro, thus generating keto-BAs and iso-BAs (49). The results showing increased absolute levels of secondary BAs, such as UDCA, 7-KDCA, DCA, and LCA in feces of the BA^+^IBS-D subgroup support the findings of BA-transforming-related genes and activities. We note that, among changed secondary BAs, UDCA and 7-KDCA exhibited higher proportions, while LCA showed a lower proportion, suggesting that *Clostridia*-rich microbiota seems more likely to prefer C7 isomerization and oxidation. Overall, the increased secondary BAs and related elevated gene expression demonstrate a changed BA biotransformation pattern for the *Clostridia*-rich microbiota of the BA^+^IBS-D patients.

BAs are natural ligands for FXR, which has been considered a master regulator of BA synthesis and secretion in the intestine and liver through negative feedback (50). Our in vitro studies found that UDCA has antagonistic effects on FXR resulting in reduced FGF15/19 production, which is in line with the finding in a recent study about UDCA in obese patients. That study showed that UDCA antagonized the intestinal feedback control system, resulting in a reduced serum FGF19 level (51). We also found that 7-KDCA, derived from microbial C7 oxidation, had no effect on *Fxr* and *Fgf19* expression in enterocytes but significantly inhibited CDCA-activated *Fgf19* expression. Further, as abundant GUDCA and TUDCA were detected in BA^+^IBS-D donors and mouse recipients, which resulted from decreased deconjugation by the *Clostridia*-rich microbiota, we also analyzed their effects on FXR. GUDCA was found to antagonize FXR, resulting in inhibition of intestinal FGF19/15 production, which is consistent with a previous study (52). In contrast with our in vivo results of an inhibition on FXR, a previous study revealed that the inhibitory effect of TUDCA on FGF19/15 expression was attributable to its antagonistic effect on FXR (52). The impact of TUDCA on FXR expression and activity requires further verification. As an excess of GCDCA was detected in sera of BA^+^IBS-D patients, which is a consequence of the decreased microbial deconjugation, we detected the potential effects of GCDCA on FXR activity. Our results showed that the *Fxr* and *Fgf19* expression by enterocytes was unchanged upon GCDCA treatment, but CDCA-activated *Fgf19* expression can be significantly attenuated. These findings indicate that there is an interaction between CDCA, the most efficacious BA ligand of FXR, and GCDCA for *Fgf19* expression. Previous studies have shown that GCDCA can activate FXR in vitro (53). Two other studies using cultures of human ileal explants also showed a stimulatory effect of GCDCA on FGF19 mRNA expression (28, 54). However, with the same dose as previously used in the former studies, Zhang et al. reported that GCDCA has no effect on FXR activation and FGF19 expression in Caco-2 cells (55). The conflicting in vitro data about the effects of GCDCA on FXR activation implies a necessity to verify the results, and in vivo tests concerning the effects of GCDCA with or without CDCA or other BAs on intestinal FGF19 production may provide a resolution.

It is known that BAs downregulate the rate-limiting genes for BA synthesis via FXR activation of SHP gene induction (50). Our studies showed that *Shp* expression was suppressed in the liver of mice colonized with *Clostridia*-rich microbiota or *C*. *scindens*. Similarly, *Clostridia*-derived TUDCA was found to reduce SHP expression in hepatocytes in vitro. Our results imply a possible inhibition of BAs derived from the *Clostridia*-rich microbiota on hepatic FXR feedback signaling. However, some of the in vitro results on FXR/SHP signaling could not be replicated in mice with BA interventions. Whether and how BAs affect hepatic FXR/SHP signaling in humans needs to be further systematically investigated. Collectively, as shown schematically in Figure 5F, our results suggest that the *Clostridia*-rich microbiota results in higher proportions of certain secondary BAs (e.g., UDCA, UDCA conjugates, and 7-KDCA) that can attenuate intestinal FXR/FGF19 signaling and thus contribute to the enhanced hepatic synthesis and fecal excretion in IBS-D patients.

Our study clarified a causal relationship between *Clostridia* bacteria and intestinal FGF19 deficiency. Antimicrobial studies in piglets showing that amoxicillin decreased abundances of *Clostridium* bacteria but enhanced animal ileal and serum FGF19 levels (56) also support such a relationship. Given that there is intestinal crosstalk between gut bacteria and BAs (22), we cannot exclude the possibility that enrichment of *Clostridia* bacteria might be secondary to the BA abnormality induced by FGF19 deficiency. Further clinical studies with an antibiotic trial in BA^+^IBS-D patients will help to clarify this issue. Recently, a bile-tolerant genera, *Bilophila*, was reported to be increased in the mouse GI lumen by TCA administration (57). It is possible that the increase in bile-tolerant bacteria, like *Escherichia* and *Bilophila*, which have been identified in BA^+^IBS-D microbial communities, occurs due to excess luminal BAs. This might explain the close relationships between several non-*Clostridia* genera and serum C4 and fecal total BAs that are present in IBS-D patients. The crosstalk between gut microbiota and excess luminal BAs should be further investigated.

The findings from this study could affect the clinical management of IBS. Previously, fecal BA analysis, or its combination with serum BA synthetic markers (C4 and FGF19), has been suggested as an auxiliary index for diagnosis of IBS-D with BAD in clinical practice, followed by ^75^Se-homocholic acid taurine scintigraphy (SeHCAT) retention and BA sequestration (58). Our findings suggest that, apart from total and primary BAs (CA and CDCA) (10, 59), secondary BAs (GUDCA, UDCA, and 7-KDCA) and their transforming *Clostridia* bacteria also have potential to be used in stratifying IBS-D populations. Further clinical studies are needed to clarify the predictive ability of specific BAs and their related bacteria in stratifying BAD in IBS-D and non–IBS-D. Our findings also suggest a novel therapeutic strategy that targets BA-transforming bacteria for treating IBS-D patients with BAD. Further, integrating gut microbiota analysis with metabolite analysis also has clinical significance for IBS management. The microbiota-based subgrouping concept for IBS has been proposed by Jeffery et al. (60). However, inconsistent microbial data from different research groups (33) make it be difficult to stratify IBS patients following the classical ecological approach (61). Gut microbial structures should be taken into consideration when differentiating the IBS subgroups, owing to their critical role in IBS pathophysiology (62). Our study integrating the structural and metabolic data revealed a specific BA-transforming microbiome in a subset of IBS-D patients, suggesting that integrating the gut microbiota analysis with the metabolic analysis is necessary, not only in clinical research but also in practice. We believe that accurate characterization of gut microbiota and its metabolites, which combine gut microbiota structural and functional features, will improve the precision of the existing symptom-based management of IBS.

One limitation of this study is that the causal relationship between the *Clostridia*-rich microbiota and BA-related phenotypes is insufficiently validated in IBS-D patients; such a relationship needs to be further verified in IBS-D patients with gut microbial modulation. Another limitation is that patients with other types of BAD were not included; *Clostridia*-rich microbiota and its stimulation of host BA synthesis/excretion needs to be further verified in IBS and non-IBS diarrheal patients.

In conclusion, the combination of human omics analysis and mechanistic experiments revealed that *Clostridia*-rich microbiota of BA^+^IBS-D patients contributes to excessive BA synthesis and excretion via enhancing proportions of specific secondary BAs that suppress intestinal FGF19 production. *Clostridia*-rich microbiota with excessive BA synthesis could be the basis for more precise pathogenic understanding and symptom management for IBS-D. Integrating the profiling of gut microbiota with metabolic features could provide a deep understanding of the microbiota-associated conditions, from physiological, pathophysiological, and therapeutic aspects, not just for IBS, but all gut microbiota–related diseases.

## Methods

### Subject recruitment and sample collection.

IBS-D adults (*n* = 345) meeting Rome IV diagnostic criteria were prospectively recruited at 2 Chinese medical clinics affiliated with the School of Chinese Medicine, Hong Kong Baptist University. Based on an approximately 30% of the pooled prevalence of the IBS-D population (10–12, 41), 91 healthy subjects as controls (HCs) were also invited. All subjects were instructed to provide fasting blood and morning first feces. They were required to stop using antibiotics, probiotics, prebiotics, and other microbiota-related supplements at least 1 month before sampling. Written consent was obtained from each subject prior to sample collection. Human specimens (serum and stools) were transported to the laboratory using dry ice and were frozen at –80°C. Details of patients’ diagnoses, subject recruitment, and sampling are described in the Supplemental Methods.

### Fecal metagenomic sequencing and data processing.

Microbial DNA was extracted from stool samples (200 mg) by the phenol/chloroform/isoamyl alcohol method (63). Fecal metagenomes were sequenced in high-quality fecal DNA samples. The DNA library was prepared using a TruSeq DNA HT Sample Prep Kit (Illumina) and sequenced by the Illumina Hiseq 2000 at BGI-Shenzhen. Removing low-quality bases and human genome left 83.59% of the high-quality sequences, and these were mapped using the published gene catalog database of the human gut microbiome (64). Microbial diversity was determined and taxa were identified as described previously (65, 66). Functional orthologs (KOs) were predicted against the Kyoto Encyclopedia of Genes and Genomes (KEGG) gene database (v79) by BLASTP (http://www.ncbi.nlm.nih.gov/blast/) with the highest scoring annotated hits. The relative abundances of phyla, genera, species, and KOs were calculated from the relative abundances of their respective genes.

### Measurement of C4 and FGF19 in serum samples.

Concentrations of FGF19 in human serum samples were tested using a commercial Human Fibroblast Growth Factor 19 Assay Kit (Thermo Fisher Scientific). The level of serum C4 was quantified by a mass spectrometry–based (MS-based) method developed by our group (67) and the detailed analytical parameters are shown in the Supplemental Methods.

### Quantification of BAs in human and rodents samples.

BA metabolites were individually extracted and quantified from human samples (serum and stools) and rodents’ specimens (liver and ileal contents) as previously described (67–70). With MS as the platform, 36 individual BAs and C4 were simultaneously quantified. Details of the chemical usage and analytical procedures are described in the Supplemental Methods. The tested BAs with their specific MRM transitions and MS/MS parameters are shown in Supplemental Table 7. Total serum or fecal BA levels of included subjects and animals were obtained by accumulation of all tested BAs.

### Animals.

Male adult C57BL/6J mice were purchased from the Laboratory Animal Services Centre of The Chinese University of Hong Kong, China. Animals were housed in rooms maintained on a 12-hour light/12-hour dark cycle with free access to a standard rodent diet and water.

### Transplantation of human microbiota in pseudo-germ-free mice.

An antibiotic cocktail (ABX) contained vancomycin (50 mg/kg), neomycin (100 mg/kg), metronidazole (100 mg/kg), amphotericin-B (1 mg/kg), and ampicillin (1 mg/mL). All antibiotics were purchased from Sigma-Aldrich. The pseudo-germ-free model was induced by 10 consecutive days of ABX administration prior to fecal microbial transplantation (FMT) (71). The demographics and BA-related quantitative traits of individual donors are shown in Supplemental Table 8. Stool samples of HC and IBS-D donors (*n* = 11–12/group) were separately prepared as microbiota PBS suspensions (50 mg/mL). ABX-pretreated mice were daily gavaged with 200 μL of microbiota suspension for 5 consecutive days. One week after FMT, GI motility and fecal consistency were measured following our previously published protocol (72). Fecal samples were collected at baseline (prior to ABX intervention), before FMT (after ABX intervention), and after FMT for monitoring bacterial density. Finally, after anesthesia, cecal contents were collected for testing BA-transforming bacterial abundances and activities. Other specimens (liver, ileum, and ileal contents) were collected to analyze BAs and/or BA-related genes and proteins. Oligonucleotide primers and analytical procedures for BA-related genes and detection of bacterial BA-transforming activity are shown in the Supplemental Methods.

### Manipulation of Clostridium species in conventional mice.

Eighteen mice (*n* = 6/group) were used for this experiment. A typical BA-transforming *Clostridium* strain (*C*. *scindens*; ATCC, 35704) was identified by its 16S ribosomal RNA sequence. The strain was cultured using Brain Heart Infusion broth (BHI, BD Biosciences) under anaerobic conditions (Bactron300 Anaerobic Chamber Glovebox, Shel Lab Inc.). After confirmation of its BA-converting activity in vitro, a *C*. *scindens* PBS suspension (1 × 10^8^ CFU/mL) was daily gavaged to 1 group of mice for 7 consecutive days. With antibacterial activity against *Clostridium* (73), vancomycin (0.1 mg/mL) was administered to another group of mice for inhibition of *Clostridium* species, with PBS as controls. BA-related bowel and metabolic phenotypes were tested after stopping bacterial manipulations as mentioned above in the FMT experiments.

### BA intervention in mice.

Forty mice were divided into 5 groups (*n* = 8/group). Following a previous study (57), each of the 4 experimental groups was separately gavaged with 50 mg/kg body weight of TCA, TCDCA, TUDCA, or their mixture (T-BAs) for 8 weeks. Saline was used as a control in the fifth group. The intervention dose and duration were as in the previous study (57). After the treatment, samples of liver and ileum were collected for analyzing genes encoding FXR, FGF19, SHP, and BA synthases (CYP7A1, CYP8B1) by real-time PCR.

### Treatment of human hepatocytes and enterocytes.

The human immortalized liver cell line L-02 was obtained from the Institute of Cell Biology, Chinese Academy of Sciences (Shanghai, China). The epithelial cell line NCI-H716 was purchased from ATCC (catalog CCL25). Following the provided instructions, L-02 and NCI-H716 cells were cultured in plastic dishes with Dulbecco’s Modified Eagle’s Medium (DMEM) and Roswell Park Memorial Institute (RPMI) 1640 Medium with 10% Gibco fetal bovine serum (Thermo Fisher Scientific), respectively. Further, vancomycin (10 μM to 400 μM) and *C*. *scindens* (live and heat-killed) were individually used to treat L-02 cells for 24 hours to test their effects on hepatic *CYP7A1* expression. Based on the EC_50_ range of BAs for FXR activation (37), L-02 and NCI-H716 cells were separately treated with 50 μM TCA, TCDCA, TUDCA, and T-BAs for 24 hours to analyze the impact of BA-taurine conjugates on expression of FXR, FGF19, SHP, CYP7A1, and CYP8B1. Moreover, GUDCA, GCDCA, GCA, CA, UDCA, and 7-KDCA were used to separately treat NCI-H716 cells for 24 hours, with and without CDCA supplementation, for evaluating their effects on expression of *FXR* and *FGF19* in enterocytes.

### Immunoblot analysis of proteins in tissues and cells.

Proteins were separately extracted from human cells for analyzing BA synthetic regulators (FXR, SHP, CYP7A1, CYP8B1 for L-02; FXR and FGF19 for NCI-H716). Accordingly, primary rabbit anti–β-actin (1: 5000, ab8227), goat anti-FXR (1:1000, ab51970), mouse anti-SHP (1:500, sc-271511), mouse anti-CYP7A1 (1:500, sc-293193), and mouse anti-FGF19 (1:500, sc-390621), purchased from Abcam or Santa Cruz Biotechnology, were used for Western blotting. Moreover, mouse hepatic or ileal tissues (50 mg) were used for protein extraction with RIPA buffer. Proteins were loaded in a 10% SDS-PAGE gel and blotted onto a PVDF membrane using the Trans-Blot Turbo Transfer system (Bio-Rad Laboratories). The membranes were blocked with Odyssey Blocking Buffer (927-50000, LI-COR Biosciences), and then incubated with primary rabbit anti–β-actin (1:5000, ab8227), mouse anti-CYP7A1 (1:500, sc-293193), or mouse anti-FGF15 (1:500, sc-514647) overnight at 4°C. After incubation with anti-rabbit and anti-mouse antibodies conjugated with IRDye 680RD or 800CW, membranes were scanned on the Odyssey Infrared Imaging System (LI-COR Biosciences) with Image Studio Lite Software (v5.2, LI-COR Biosciences).

### Statistics.

The software package R 3.4.3 (https://www.r-project.org/) was used to perform the Shapiro-Wilk test for judging the distribution of human total fecal BAs. The relationships of total fecal BAs with other biochemical and bacterial characteristics were analyzed by Spearman’s correlation based on Prism 7 (GraphPad Software). Differential taxa and BA-transforming genomes were analyzed with the Benjamin-Hochberg method. Variations in clinical quantitative traits, metabolites, and genes were analyzed by the nonparametric Kruskal-Wallis test for comparison of multiple groups, while the Mann-Whitney test was employed for comparison of 2 groups. Statistically significant differences were defined as *P* < 0.05.

### Data availability.

Fecal metagenomic sequencing reads can be downloaded from CNGB Nucleotide Sequence Archive (https://db.cngb.org/cnsa/) under accession number CNP0000334.

### Ethics approval.

The clinical study received approval from the Committee on the Use of Human & Animal Subjects in Teaching & Research at Hong Kong Baptist University (HASC/15-16/0300 and HASC/16-17/0027). Animal experiments followed the Animals Ordinance guidelines, Department of Health, Hong Kong SAR, and reporting of in vivo experiments also followed ARRIVE guidelines.

## Author contributions

WJ, XF, and ZB jointly designed this study. WJ and ZB supervised the study and revised manuscript. HEN and AL contributed project discussion and gave comments. ZC provided bile acid standards and gave critical comments for the study. LZ, WY, YC, and FH drafted the manuscript. The order of the 4 co–first authors was assigned following the contribution of the affiliations they belong to. LZ, WY, LL, LZ, ZN, CL, and HX collected clinical samples and performed tests of clinical samples. LZ, WY, and LL performed bacterial experiments. WY and FH tested the effects of bile acids on FXR signaling in vivo and in vitro. ZR and DC provided support for animal experiments and protein assays. TH provided guidance in data analysis and the R software. LLDZ, WL, CC, ZY, XZ, and ZB recruited IBS patients. YC, LH, QQ, XS, RH, and SS were responsible for fecal DNA extraction, sequencing, and metagenomic data analysis.

## Supplementary Material

Supplemental data

Supplemental tables 1-8

## Figures and Tables

**Table 1:**
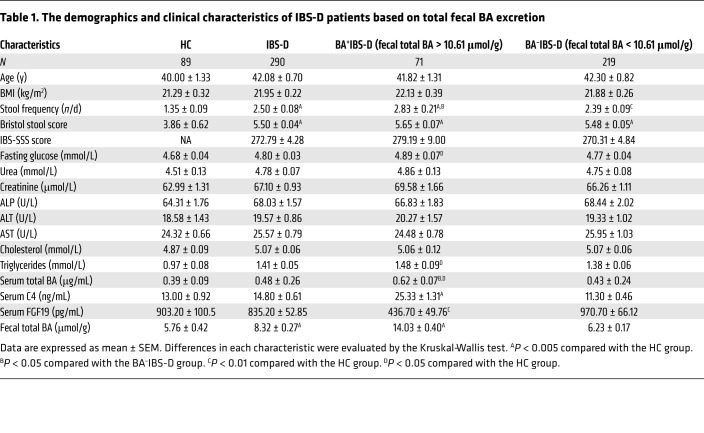
The demographics and clinical characteristics of IBS-D patients based on total fecal BA excretion

## Version 1 12/09/2019

Electronic publication

## Version 2 01/02/2020

Print issue publication

## Version 3 03/27/2024

Added supplemental tables file
