# Supplementary material for: A Clostridia-rich microbiota enhances bile acid excretion in diarrhea-predominant irritable bowel syndrome
Source: J Clin Invest. 2019 Dec 9;130(1):438–50. doi: 10.1172/JCI130976 (PMC6934182; doi:10.1172/JCI130976)
Supplement: Supplemental data [file jci-130-130976-s007.pdf]

Supplementary materials

A *Clostridia*-rich microbiota enhances bile acid excretion in diarrhea-predominant irritable bowel syndrome

Ling Zhao[1†], Wei Yang[1†], Yang Chen[2†], Fengjie Huang[3†], Lin Lu[1], Chengyuan Lin[1], Tao Huang[1], Ziwan Ning[1], Lixiang Zhai[1], Linda LD Zhong[4], Waiching Lam[4], Zhen Yang[4], Xuan Zhang[4], Chungwah Cheng[4], Lijuan Han[2], Qinwei Qiu[2], Xiaoxiao Shang[2], Runyue Huang[2], Haitao Xiao[5], Zhenxing Ren[6], Dongfeng Chen[6], Silong Sun[7], Hani El-Nezami[8], Zongwei Cai[9], Aiping Lu[4], Xiaodong Fang[2,6,7*], Wei Jia[3,10*], and Zhaoxiang Bian[1,4*]

[1]Institute of Brain and Gut Research, School of Chinese Medicine, Hong Kong Baptist University, Hong Kong SAR, China
[2]Second Affiliated Hospital of Guangzhou University of Chinese Medicine, Guangzhou, China
[3]Shanghai Key Laboratory of Diabetes Mellitus and Center for Translational Medicine, Shanghai Jiao Tong University Affiliated Sixth People's Hospital, Shanghai, China
[4]Chinese Medicine Clinical Study Center, School of Chinese Medicine, Hong Kong Baptist University, Hong Kong SAR, China
[5]School of Pharmaceutical Sciences, Health Science Center, Shenzhen University, Shenzhen, China
[6]College of Basic Medicine, Guangzhou University of Chinese Medicine, Guangzhou, China
[7]BGI Genomics, BGI-Shenzhen, Shenzhen, China
[8]School of Biological Sciences, Faculty of Science, The University of Hong Kong, Hong Kong SAR, China
[9] School of Chemistry, Hong Kong Baptist University, Hong Kong SAR, China
[10]Cancer Biology Program, University of Hawaii Cancer Center, Hawaii, USA

Supplementary materials include

Supplementary methods
Supplementary figures include
Figure. S1. Alteration of serum bile acid profile in IBS-D patients.
Figure. S2. Fecal microbial structures among control and IBS-D subjects.
Figure. S3. Changed luminal BA transformation associated with BA synthetic regulation in mouse with transplantation of IBS-D fecal microbiota.
Figure. S4. The effects of *Clostridium* species on ileal BA feedback control in vivo and hepatic synthetase in vitro.
Figure. S5. Effects of taurine-conjugated BAs on protein expressions of FXR, SHP, CYP7A1 and CYP8B1 in hepatocytes.

Supplementary methods

Subject recruitment and sampling

IBS-D patients were recruited through the advertisements or press released in local newspaper. The detail criteria used for patient recruitment are shown as follows:

Inclusion criteria: IBS-D patients were recruited on if they fulfilled the following criteria: 1) meet of Rome IV criteria, including recurrent abdominal pain on average at least 1d/week in the last 3 months; 2) appearance of stool form with at least 25% of loose or watery stools and fewer than 25% of hard stools based on Stool Bristol Score; 3) IBS Symptom Severity Scale (IBS-SSS) over than 75 points at baseline; 4) age of 18 to 65 years; 5) normal colonic evaluation with 5 years by examination of colonoscopy or barium enema; 6) Written informed consent.

Exclusion criteria: Patients were excluded if they have one or more of follows: 1) pregnancy or breast-feeding; 2) medical history of inflammatory bowel diseases, carbohydrate malabsorption, hormonal disorder, known allergies to food additives, and/or any other serious diseases; 3) surgical histories of gallbladder removal, GI tract, and cerebral cranium; 4) having parasitic infections; 5) having suicidal ideas or attempts or aggressive behavior; 6) use of medications known to influence gastrointestinal function, blood pressure and fat.

The sample size of matched healthy controls was expected referred to the around 30% of pooled prevalence of excess total BA excretion in IBS-D population. The recruiting criteria for healthy population is shown as follows:

Inclusion criteria: 1) age of 18–65 years (inclusive); 2) no medical history of metabolic disorders, cardiovascular diseases, neurodegenerative diseases, and gastrointestinal diseases; 4) normal hepatic, renal, and bowel functions within 3 years; 4) no drug taken history for chronic diseases, metabolic diseases, cardiovascular and cerebrovascular diseases, psychiatric illness, disease of immune system, and other serious diseases within 1 year; 5) written informed consent.

Exclusion criteria: 1) pregnancy or breast-feeding; 2) surgical histories of gallbladder removal, GI tract, and cerebral cranium; 3) having parasitic infections; 4) use of medications known to influence gastrointestinal transit, blood pressure and fat.

A questionnaire of IBS symptoms contained IBS-SSS, defecation frequency within a day and Bristol Stool Form Scale was required to complete when the interview with physicians. Fecal consistency of individuals was assessed by the score of the Bristol Stool Form Scale. Meanwhile, another dietary questionnaire that recorded complete diet information and dietary habits within past three months was also required to be completed upon subject recruitment and, those with specific dietary habits, such as alcohol consumption or a completely vegetable-based diet, were excluded as well. Participants were instructed to provide fasting blood samples and morning first feces for analyzing 1) serum biochemical indices and stool culture test (conducted by Chan & Hou Medical Laboratories Ltd, Hong Kong); 2) bile acid (BA) profiles and metagenomics. They were also required to stop using antibiotics, probiotics, prebiotics and other microbiota-related supplements at least one month before sampling.

Targeted profiling of bile acids and C4 based on UPLC/MS

Chemicals and reagents. A total of 36 BA metabolites, including cholic acid (CA), ω-muricholic acid (ωMCA), α-muricholic acid (αMCA), β-muricholic acid (βMCA), hyocholic acid (HCA), hyodeoxycholic acid (HDCA), chenodeoxycholic acid (CDCA), ursodeoxycholic acid (UDCA), deoxycholic acid (DCA), isodeoxycholic acid (IsoDCA), isolithocholic acid (IsoLCA), 7-ketodeoxycholic acid (7-KDCA), 6-ketolithocholic acid (6-KLCA), 7-ketolithocholic acid (7-KLCA), 12-ketolithocholic acid (12-KLCA), dehydrocholic acid (DHCA), lithocholic acid (LCA), allo-cholic acid (ACA), 23-nordeoxycholic acid (23-NDCA), glycocholic acid (GCA), glycochenodeoxycholic acid (GCDCA), glycodeoxycholic acid (GDCA), glycolithocholic acid (GLCA), glycodehydrocholic acid (GDHCA), glycohyodeoxycholic acid (GHDCA), glycohyocholic acid (GHCA),

glycoursodeoxycholic acid (GUDCA), taurocholic acid (TCA), taurodeoxycholic acid (TDCA), tauroursodeoxycholic acid (TUDCA), taurohyocholic acid (THCA), taurohyodexoycholic acid (THDCA), taurochenodeoxycholic acid (TCDCA), taurolithocholic acid (TLCA) were purchased from Sigma-Aldrich (St. Louis, MO, USA). Tauro-α-muricholic acid (TαMCA) and Tauro-β-muricholic acid (TβMCA) were purchased from Santa Cruz Biotechnology (Santa Cruz, CA, USA). An isotopic BA deoxycholic acid-2,2,4,4-d4 (DCA-d4), served as internal standard and was obtained from CDN isotopes (Pointe-Claire, Quebec, Canada). HPLC grade organic reagents for mass spectrometric analysis were purchased from Sigma-Aldrich (St. Louis, MO, USA).

Stock solution and calibration curve preparation. All BA chemical standards were separately dissolved in methanol as stock solution with a concentration of 5 mg/ml. A mixed stock solution was obtained after mixing individual standard stock solution. Diluting stock solutions in methanol, the working solution were prepared at a series concentration of 0.020, 0.102, 0.512, 2.56, 12.8, 64, 320, 1600, 8000, and 40000 ng/ml for individual BAs, while at a series concentration of 0.064, 0.32, 1.6, 8, 40, 200, 1000, and 5000 ng/ml for serum C4. The standard curves and regression coefficients were gained based on IS adjustment. The signals of each BA metabolites were found in individual measured ranges.

UPLC/TQ-MS condition. An ultra-high-performance liquid chromatography (Agilent UHPLC 1290, USA) coupled with a triple-quadrupole mass spectrometer (Agilent QQQ-MS 6438, USA) was applied for bile acid analysis. Though a single 26-min acquisition with positive/negative ion switching, bile acid metabolites (under ESI-) and C4 (under ESI+) were simultaneously quantified in multiple reaction monitoring (MRM) mode. Sample injection and flow rate were set at 2 μL and 0.35 ml/min for each sample, respectively. Bile acid metabolites were separated using an ACQUITY BEH C18 column (1.7 μm, 100mm × 2.1 mm) with a linear gradient of 0.1% formic acid (FA) in water (A)

and 0.1% FA in acetonitrile (B). The gradient program was: 25% to 40% B for the first 6 min, 40% to 70% B for 14 min, 70% to 100% B for 0.1 min, held at 100% B for 2.9 min, then re-equilibration at 25% B for 0.1 min, and held at 25% B for 2.9 min. The column temperature was maintained at 45℃. The capillary voltage of mass spectrometer was 3.5 kV and 4 kV in positive and negative modes. The acquisition data was analyzed using Agilent MassHunter Workstation Software for peak integration, calibration equations and quantification of individual BAs.

Measurement of bacterial BA-transforming activities

The BA-transforming activities of gut bacteria isolated from human feces or mouse cecal contents were assessed in vitro by quantifying the BA products and substrates for each transforming action using LC/MS. The concentration of supplemented BA substrates was referred to the level of total BAs in feces of human beings. In details, mixed bacteria were individually extracted from human feces using 10-fold sterile PBS with differential centrifugation. A volume of 1mL of bacterial reaction mixture, containing bacterial extracts (diluted as 0.1 of the final OD600 value), BHI medium (BD Biosciences) and 5 mM BA substrate (GCDCA, CDCA or CA), were prepared and shaken continuously at 37°C for 24 hours. Then, BA metabolites were extracted from the culture medium using 3-fold methanol for LC/MS-based quantitative analysis. The ratio of CDCA to GCDCA represented the level of bacterial deconjugation, the ratio of UDCA to CDCA and DCA to CA were used for evaluating bacterial 7-HSDH and 7α-dehydroxylating levels, respectively. For mouse BA-transforming activity analysis, Mixed bacteria were isolated from cecal contents, and the bacterial reaction mixture with 5 mM BA substrate (TCA, CDCA or CA) were prepared as mentioned above. The level of bacterial deconjugation was evaluated by the ratio of TCA to CA, while bacterial 7-HSDH and 7α-dehydroxylating levels were tested with the ratio of UDCA to CDCA and the ratio of DCA to CA, respectively.

Quantitative real-time PCR analysis of bacterial and host genes

Total DNA was extracted from cecal contents (100 mg) of FMT or *Clostridium*-treated mice for specific bacteria analysis using real-time PCR detection. Briefly, TB Green Premix detection kits (Cat# RR820B, Takara) were purchased for preparation of qPCR reaction mixture, containing DNA template (50ng), 0.5μM each DNA oligonucleotide primers (Invitrogen), and TB Green Master Mix (2X). Bacterial genes were amplified using a real-time PCR cycler (ViiA^{TM} 7Dx Instrument, Applied Biosystems). The relative levels of each bacterium from each DNA samples was normalized to the total bacterial expression. Moreover, total RNA samples were individually isolated from the hepatic and ileum tissues of experimental mice using TissueLyzer (Qiagen) with Trizol reagent (Invitrogen). The cDNAs were produced from RNA samples using the SuperScript® First-Strand synthesis system (Invitrogen). Quantitative real-time PCR detection was performed on the ViiA™ 7 Real-Time PCR System, and analysis of target gene expression was processed with the $\Delta\Delta C_T$ method. Oligonucleotide primers for bacteria and host genes related to BA metabolism are summarized as below.

**Origins**	**Targeted genes**	**Sequences (5’→3’)**
**Bacteria**	*Total bacteria*	F: GTGSTGCAYGGYTGTCGTCA
		R: ACGTCRTCCMCACCTTCCTC
	Bacteroidetes	F: GGARCATGTGGTTTAATTCGATGAT
		R: AGCTGACGACAACCATGCAG
	Firmicutes	F: GGAGYATGTGGTTTAATTCGAAGCA
		R: AGCTGACGACAACCATGCAC
	Clostridium Cluster XIVa	F: AAATGACGGTACCTGACTAA
		R: CTTTGAGTTTCATTCTTGCGAA
	Clostridium scindens	F: GCAACCTGCCTTGCACT
		R: ACCGAATGGCCTTGCCA
**Mouse**	Cytochrome P450 7A1 (CYP7A1)	F: AGCAACTAAACAACCTGCCAGTACTA
		R: GTCCGGATATTCAAGGATGCA
	Cytochrome P450 27A1 (CYP27A1)	F: GCCTCACCTATGGGATCTTCA
		R: TCAAAGCCTGACGCAGATG
	Cytochrome P450 8B1 (CYP8B1)	F: GGCTGGCTTCCTGAGCTTATT
		R: ACTTCCTGAACAGCTCATCGG
	25-hydroxycholesterol 7α-hydroxylase (CYP7B1)	F: TAGCCCTCTTTCCTCCACTCATA
		R: GAACCGATCGAACCTAAATTCCT
	Farnesoid X Receptor (FXR)	F: TGTGAGGGCTGCAAAGGTT
		R: ACATCCCCATCTTGGAC

	Fibroblast growth factor receptor 4 (FGFR4)	F: GCCTCCGACAAGGATTTGGCA
		R: GAGTGCAGACACCCAGCAGGT
	Beta-Klotho (KLB)	F: CAGAGAAGGAGGAGGTGAGG
		R: CAGCACCTGCCTTAAGTTGA
	Small heterodimer partner (SHP)	F: CGATCCTCTTCAACCCAGATG
		R: AGGGCTCCAAGACTTCACACA
	Fibroblast growth factor 15 (FGF15)	F: ACGTCCTTGATGGCAATCG
		R: GAGGACCAAAACGAACGAAATT
	Apical sodium dependent bile acid transporter (ASBT)	F: ACCACTTGCTCCACACTGCTT
		R: CGTTCCTGAGTCAACCCACAT
	Multidrug resistance associated protein 2 (MRP2)	F: CTGAGTGCTTGGACCAGTGA
		R: CAAAGTCTGGGGGAGTGTGT
	Multidrug resistance associated protein 3 (MRP3)	F: CGCTCTCAGCTCACCATCAT
		R: GGTCATCCGTCTCCAAGTCA
	Organic solute transporter α (Ostα)	F: TGTTCCAGGTGCTTGTCATCC
		R: CCACTGTTAGCCAAGATGGAGAA
	Organic solute transporter β (Ostβ)	F: GATGCGGCTCCTTGGAATTA
		R: GGAGGAACATGCTTGTCATGAC
	Beta-actin (β-actin)	F: ACCTGACAGACTACCTCATGAAGA
		R: TCATGGATGCCACAGGATTCCATA

Supplementary figures

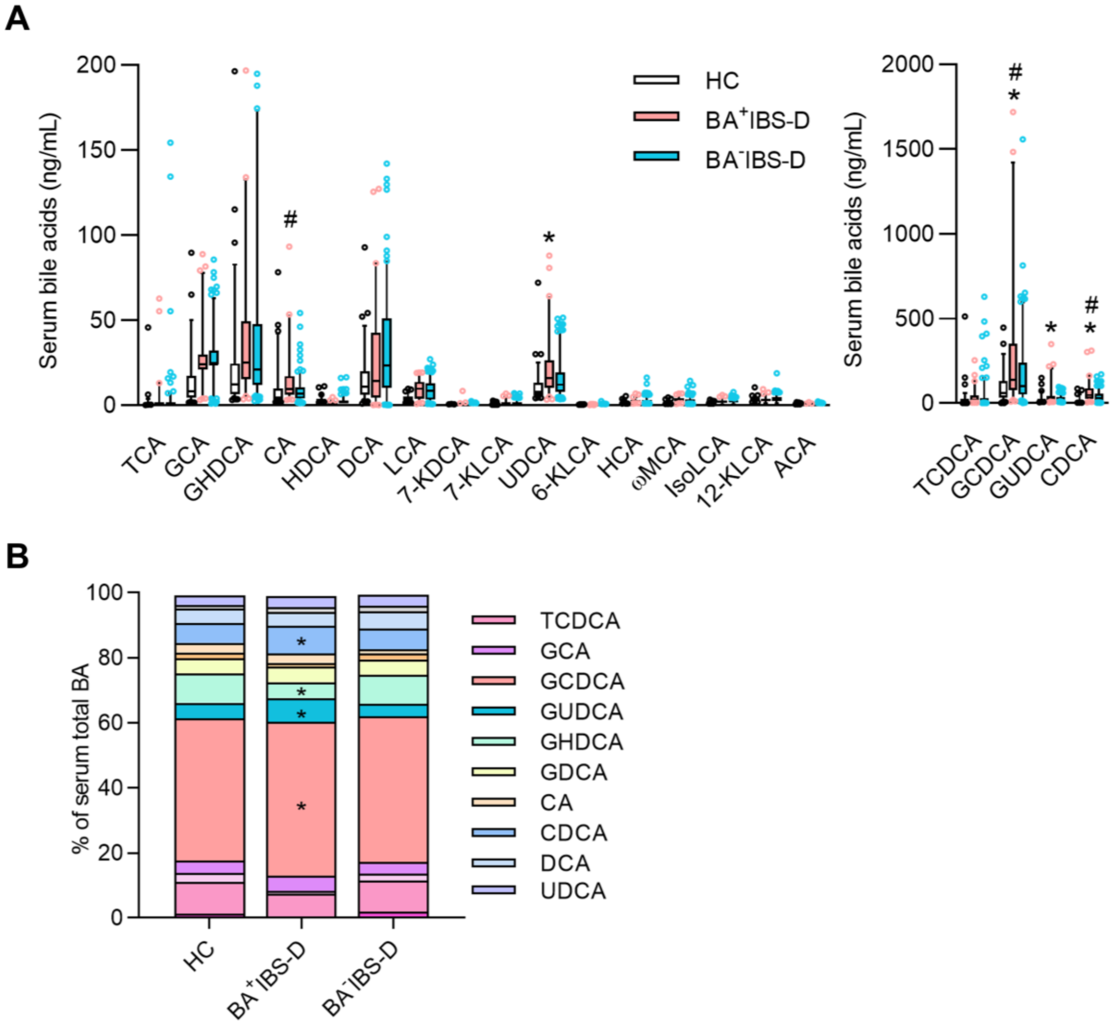

Figure S1. Alteration of serum bile acid profile in IBS-D patients. (A) Absolute contents of serum dominant BA metabolites. (B) Proportions of serum dominant BAs. Only BAs with over 2% of total BA are shown in the legend. Serum BA individuals were analyzed by LC/MS. Absolute amounts of BA metabolites are expressed with the whisker chart using 5th-95th percentile values, relative level of BA individual is plotted with mean value. Comparison of BA metabolites is analyzed by the Kruskal-Wallis test. Statistical significance is expressed by *, p<0.05 compared with the HC group; #, p<0.05 compared with the BA^{-}IBS-D group.

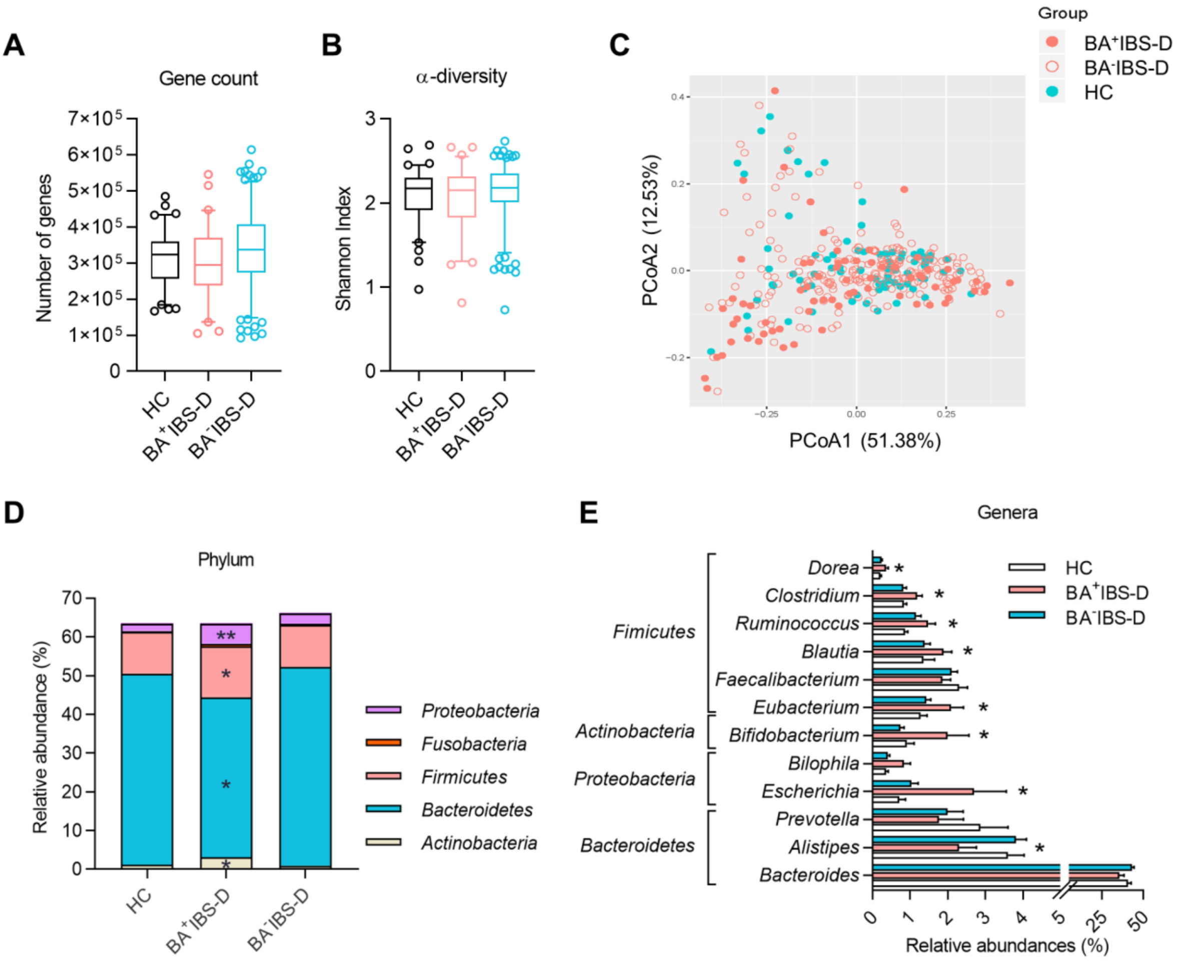

Figure S2. Fecal microbial structures among control and IBS-D subjects. (A) The total gene count obtained from the metagenomic dataset of human fecal samples. (B) Microbial α-diversity measured by the Shannon index. (C) Principle component analysis of human microbial communities based on Bray-Curtis dissimilarity. (D, E) The relative abundances of dominant phyla and genera identified from human fecal microbiomes. Differential taxa among three groups are analyzed with the Benjamin-Hochberg method. The whisker chart is plotted by the 5th-95th percentile values and, bar chart is shown as mean ± SEM. Statistical significance is expressed by *, $p<0.05$; **, $p<0.001$ compared with the HC group. Spearman's correlation is performed in GraphPad Prism 7, and statistical significance is set as *, $p < 0.05$.

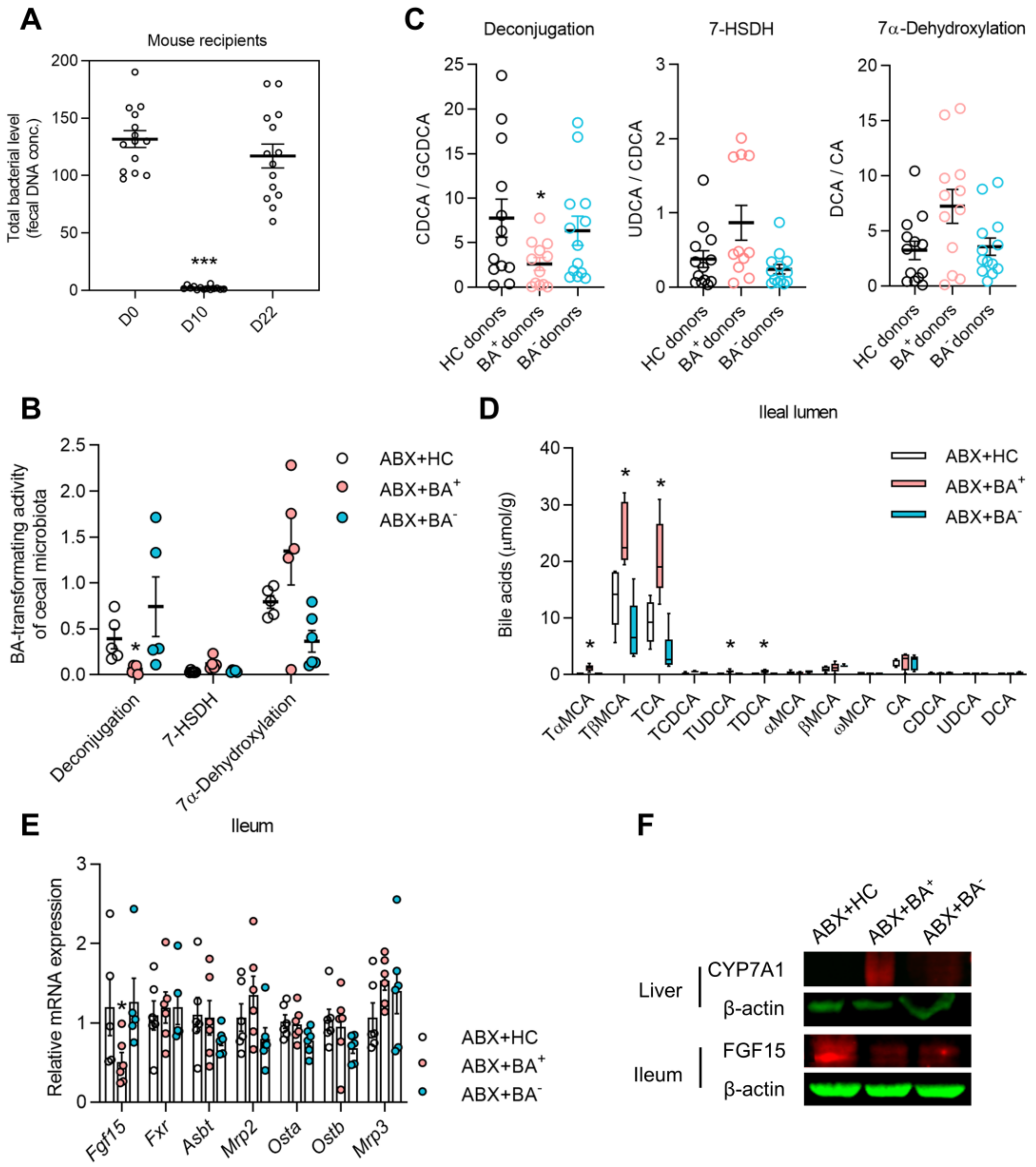

Figure S3. Changed luminal BA transformation associated with BA synthetic regulation in mouse with transplantation of IBS-D fecal microbiota. (A) Dynamic alteration of cecal total bacterial counts throughout the FMT experiment. (B, C) Bacterial BA-transforming levels of donors and mouse recipients. Each BA-transforming activity was measured by the ratio of the BA product to substrate. (D) The BA profile of the ileal contents in mouse recipients. (E) Relative gene expression of ileal proteins related to BA feedback and transport in mouse recipients. (F) Protein expressions of hepatic CYP7A1 and ileal FGF19. The dot plot is presented as mean ± SEM and, the whisker chart is plotted by the 5th-95th percentile values. Statistics are analyzed by the Kruskal-Wallis test. Statistical significance is expressed by *, p<0.05; ***, p<0.005 compared with individual control group.

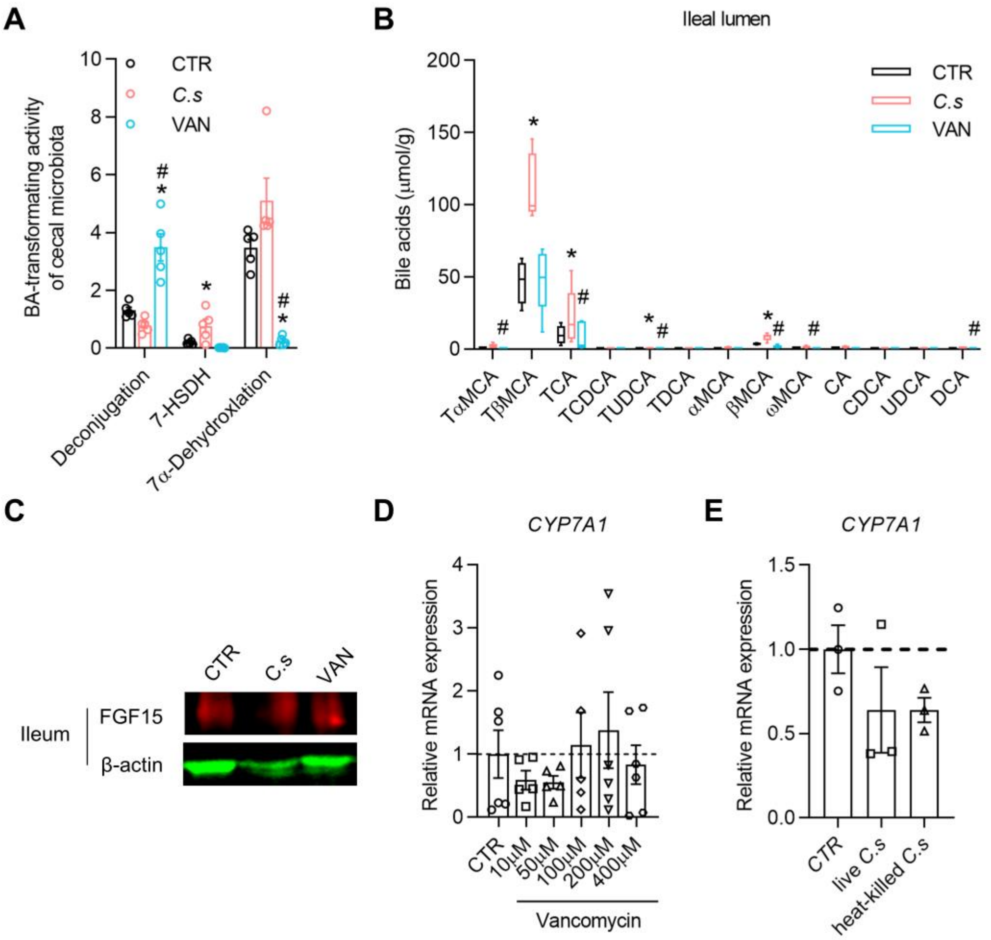

Figure S4. The effects of *Clostridium* species on ileal BA feedback control in vivo and hepatic synthetase in vitro. (A) Cecal microbial BA-transforming levels in mice with manipulation of *Clostridium* species. (B) Luminal BA profile of mouse ileum. (C) Protein expression of ileal FGF15. (D, E) Gene expressions of CYP7A1 when culturing hepatocytes in presence of vancomycin, live or heat-killed *C. scindens*. The dot plot is presented as mean ± SEM and, the whisker chart is plotted by the 5th-95th percentile values. Statistics are analyzed using the Kruskal-Wallis test. Statistical significance is expressed by *, $p<0.05$ compared with the control group; #, $p<0.05$ compared with the *C. scindens* group.

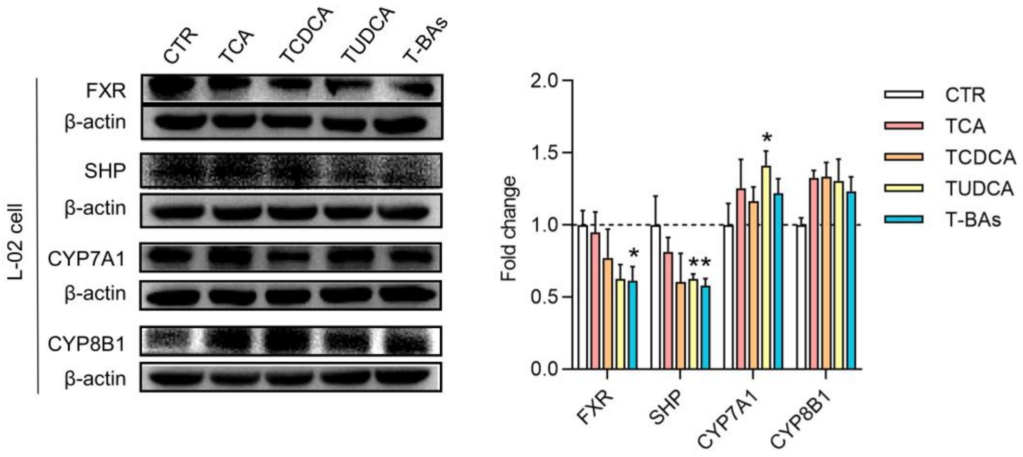

Figure S5. Effects of taurine-conjugated BAs on protein expressions of FXR, SHP, CYP7A1 and CYP8B1 in hepatocytes. Differential proteins are presented as mean ± SEM, statistics are analyzed with the Kruskal-Wallis test. Statistical significance is expressed by *, p<0.05; **, p<0.01 compared with the control group.
